# A response‐adaptive randomization procedure for multi‐armed clinical trials with normally distributed outcomes

**DOI:** 10.1111/biom.13119

**Published:** 2019-09-19

**Authors:** S. Faye Williamson, Sofía S. Villar

**Affiliations:** ^1^ Department of Mathematics and Statistics Lancaster University Lancaster UK; ^2^ MRC Biostatistics Unit, School of Clinical Medicine University of Cambridge Cambridge UK

**Keywords:** adaptive designs, continuous endpoint, dichotomization, Gittins index, missing data, unknown variance

## Abstract

We propose a novel response‐adaptive randomization procedure for multi‐armed trials with continuous outcomes that are assumed to be normally distributed. Our proposed rule is *non*‐myopic, and oriented toward a patient benefit objective, yet maintains computational feasibility. We derive our response‐adaptive algorithm based on the Gittins index for the multi‐armed bandit problem, as a modification of the method first introduced in Villar *et al*. (*Biometrics*, 71, pp. 969‐978). The resulting procedure can be implemented under the assumption of both known or unknown variance. We illustrate the proposed procedure by simulations in the context of phase II cancer trials. Our results show that, in a multi‐armed setting, there are efficiency and patient benefit gains of using a response‐adaptive allocation procedure with a continuous endpoint instead of a binary one. These gains persist even if an anticipated low rate of missing data due to deaths, dropouts, or complete responses is imputed online through a procedure first introduced in this paper. Additionally, we discuss how there are response‐adaptive designs that outperform the traditional equal randomized design both in terms of efficiency and patient benefit measures in the multi‐armed trial context.

## 1 INTRODUCTION

Response‐adaptive randomization (RAR) has been widely developed ever since the idea was first suggested by Thompson (1933) (Hu and Rosenberger, 2006). The usual motivation behind RAR is to achieve a patient benefit objective, for example, to reduce exposure to inferior treatments by skewing the allocation toward superior treatments based on observed responses. Incorporating such an objective into a trial design is particularly important when the disease under study is rare—in which case a substantial proportion of patients in the population will be included in the trial—and when an inferior treatment could result in a fatal outcome.

Despite the vast array of RAR procedures proposed in the literature, most of them: (a) assume *binary* responses, (b) are defined for trials with only *two treatments*, and (c) are *myopic*. However, many clinical trials have *continuous* primary outcomes and include more than two (multiple) arms. Wason and Trippa (2014) report that 39% of all multi‐arm clinical trials published in four major medical journals during 2012 had normally distributed primary outcomes. Although most RAR procedures for binary responses are not easily extended to the continuous case, particularly those based on urn models (Atkinson and Biswas, 2013), several RAR procedures for continuous outcomes have been proposed (eg, Zhu and Hu, 2009); a review of these can be found in chapter 4 of Atkinson and Biswas (2013), and Biswas and Bhattacharya (2016). Moreover, a “shortage of RAR methodology to handle cases with multiple treatments” (Zhang *et al*., 2011) persists, despite the fact that RAR has the greatest potential for efficiency and patient benefit gains in multi‐armed trials (Berry, 2011), which considerably limits their use in practice.

Furthermore, almost all procedures in the RAR literature (for binary or continuous outcomes) use only *past* observations (allocations and responses) to influence the decision for the next patient, without considering the number of patients remaining to be treated (inside or outside the trial) or the information they could provide. Such *myopic* strategies are not optimal in general (Berry and Fristedt, 1985). An optimal approach, in terms of patient benefit, is based on the multi‐armed bandit problem (MABP) which considers *all* possible sequences of trial observations, and the sequence that maximizes patient response is selected (Villar *et al*., 2015a). As a result, the traditional dynamic programming approach used to solve the MABP is much more computationally intensive than myopic procedures, which is the predominant reason why the latter have been favored in the literature. Recent work proposing *non*‐myopic bandit‐based RAR procedures for binary responses includes Villar *et al*. (2015b), Williamson *et al*. (2017), and Villar and Rosenberger (2018). We will refer to *non*‐myopic procedures as *forward‐looking* hereafter to be consistent with the terminology used in previous papers.

Examples of forward‐looking adaptive allocation rules for continuous endpoints relevant to this paper are Coad (1991b), Wang (1991a), and Smith and Villar (2018), all of which use the Gittins index for normally distributed outcomes. However, the main limitation of these designs from a clinical trials perspective is their deterministic nature. Randomization is essential in order to remove various sources of bias and it additionally provides a basis for inference (Rosenberger and Lachin, 2015).

Motivated by the above considerations, we propose a novel bandit‐based allocation rule that (a) applies to continuous outcomes, assumed to be normally distributed; (b) applies when the outcome variance is assumed unknown; (c) is defined for multi‐armed trials; (d) is forward‐looking and thus is oriented toward a patient benefit objective; (e) is computationally feasible, and (f) is randomized. Additionally, we investigate the impact on patient benefit of dichotomizing a continuous endpoint, which is a widely adopted approach in clinical research that has received considerable attention in the literature (Royston *et al*., 2006). A common reason for this practice is to deal with complete responses and missing data (due to death or dropout, for example) since these naturally fall into success and failure categories, respectively. However, dichotomization comes at an efficiency cost (either a reduced power or larger sample size) (Lavin, 1981; Wason *et al*., 2011).

Dealing with complete responses and missing data poses an extra challenge that is exclusive to the implementation of RAR in a trial. The imputation method suggested in Karrison *et al*. (2007), which is the only one that has shown moderate uptake in practice (Wason and Jaki, 2016), imputes unobserved responses using the distribution of data collected at the *end* of the trial and therefore, the imputed data cannot be used to perform any adaptations. In this paper, we suggest a simple modification of the procedure by Karrison *et al*. (2007) which permits the use of RAR to allocate patients dynamically during the trial.

In Section 2, we present our forward‐looking rule for continuous endpoints with unknown variance using a simple example to illustrate its implementation. In Section 3, we report extensive comparative simulation studies in the context of a real phase II cancer trial. We discuss the costs of dichotomization in Section 4, and present our method to accommodate missing data due to deaths, dropouts and complete responses in Section 5. We draw conclusions in Section 6.

## 2 THE FORWARD‐LOOKING GITTINS INDEX RULE FOR CONTINUOUS ENDPOINTS

We now define a RAR procedure for continuous endpoints, assumed to be normally distributed, which augments the Forward‐Looking Gittins Index (FLGI) rule proposed in Villar *et al*. (2015b) for binary endpoints. Following the notation in that paper, we consider a clinical trial that will test the effectiveness of K experimental treatments against a control treatment on a sample of T patients, with K and T fixed. Patients are labeled by t (t=1,…,T) and treatments by k (k=0,…,K), where k=0 denotes the control. The response of patient t allocated to treatment k is a random variable denoted by $Y_{k,t}$, now assumed to follow a normal distribution, $Y_{k,t}∼N\left(\mu_{k},\sigma_{k}^{2}\right)$. Without loss of generality, we also assume that a *larger* response is desired and that $\sigma_{k}^{2}$ is unknown.

In order to derive our FLGI rule, we need to obtain the Gittins index for the MABP associated with this trial design problem. A detailed explanation of the problem's assumptions and its exact formulation appears in Supporting Information Appendix A. The Gittins index for a treatment with posterior mean ${ỹ}_{k,t}$ and posterior standard deviation ${\tilde{s}}_{k,t}$, after having observed $n_{k,t}$ responses from treatment $k,G\left({ỹ}_{k,t},{\tilde{s}}_{k,t},n_{k,t}\right)$, can be written as

(1) $$G\left({ỹ}_{k,t},{\tilde{s}}_{k,t},n_{k,t}\right)={ỹ}_{k,t}+{\tilde{s}}_{k,t}G\left(0,1,n_{k,t}+2,d\right),$$

where $G\left(0,1,n_{k,t}+2,d\right)$ denotes the Gittins index value of a standardized bandit problem with posterior mean 0, posterior standard deviation 1, $n_{k,t}$ observations, an implicit (prior) sample size of 2 (refer to Supporting Information Appendices A and C), and discount factor 0≤d<1. In this paper, we choose d as recommended in Wang (1991b) (Supporting Information Appendix B provides further details).

Notice that in this case we have two unknown parameters, $\mu_{k}$ and $\sigma_{k}^{2}$, which we assume have the hierarchical conjugate priors $\mu_{k}|\sigma_{k}^{2}∼N(0,\sigma_{k}^{2}/2)$ and $\sigma_{k}^{2}∼IG\left(1/2,1/2\right)$, that is, the normal‐inverse‐gamma joint prior $\left(\mu_{k},\sigma_{k}^{2}\right)∼NIG\left(0,2,1/2,1/2\right)$, when $n_{k,t}=0$. The choice of prior and its effect on performance measures is explored in Supporting Information Appendix C. As in Smith and Villar (2018), we implement the solution in (1) at a very low computational cost by calculating the values of $G\left(0,1,n_{k,t}+2,d\right)$ in advance and interpolating from the tables in Gittins *et al*. (2011). Details on how to compute these indices, first computed by Jones (1975), can be found in chapters 7 and 8 of Gittins *et al*. (2011).

In order to derive a response‐adaptive rule that will sequentially randomize the next b patients among the K+1 treatments at stage j (j=1,…, J), given the data up to and including block j−1, according to what the Gittins index rule would do, we assume that patients are enrolled in groups of size b over J stages, so that J×b=T. Using (1) and the Gittins index rule, which states that it is optimal to allocate the treatment with the highest index value (breaking ties at random), we can compute the FLGI probabilities for the case of a normally distributed endpoint (with unknown variance) using equation (3) in Villar *et al*. (2015b). The main difference here is that the optimal action probabilities in equation (3) of Villar *et al*. (2015b) can no longer be matched to the probabilities of the (binary) outcome and must be computed for different ranges of the continuous outcome.

### 2.1 Example

To illustrate the proposed rule, we derive the FLGI probabilities for the simplest possible case of a two‐arm trial testing a control treatment (k=0) against an experimental treatment (k=1) with a block of size two (b=2).

For both k, we assume the following hierarchical (conjugate) prior structure at the start of the trial: $\mu_{k}|\sigma_{k}^{2}∼N(0,\sigma_{k}^{2}/2)$ and $\sigma_{k}^{2}∼IG\left(1/2,1/2\right)$, so that $\left(\mu_{k},\sigma_{k}^{2}\right)∼NIG\left(0,2,1/2,1/2\right)$. Suppose further that both patients are randomly allocated to the control treatment in the first block of the trial, resulting in responses $y_{0,1}=3.1$ and $y_{0,2}=-0.4$. Thus, the three relevant parameters required to obtain the corresponding Gittins index for the *control* treatment are: the posterior mean ${ỹ}_{0,2}=0.675$, the posterior standard deviation ${\tilde{s}}_{0,2}=1.727$, and the number of observations $n_{0,2}=2$. For the *experimental* treatment, the relevant parameters are: ${ỹ}_{1,2}=0,{\tilde{s}}_{1,2}=1$, and $n_{1,2}=0$. From Equation (1), setting d=0.995 and using Table S1, the Gittins index for the control and experimental treatment, respectively, is $G_{0}\left(0.675,1.727,2\right)=0.675+1.727\times 1.8126=3.805$ and $G_{1}\left(0,1,0\right)=0+1\times 65.5848=65.585$.

**Figure 1:**
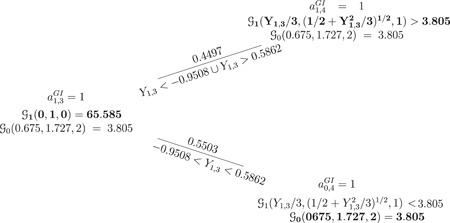
The FLGI rule and a probability tree of all trial histories using the Gittins index rule when K+1=2,b=2,d=0.995, the outcome $Y_{k,t}$ is normally distributed with unknown mean and variance, and parameters $\left({\tilde{y}}_{k,3},{\tilde{s}}_{k,3},n_{k,3}\right)$ are given by (0.675,1.727,2) for k=0 and (0,1,0) for k=1. Bold text indicates the allocated treatment under the Gittins index rule $\left\{a_{k,t}^{GI}\right\}$. Note that the FLGI probabilities in this case are 0.7249 and 0.2751 for the experimental and control arm respectively. (For simplicity of the illustration, we have omitted the branch corresponding to the cases $Y_{1,3}=-0.9508$ or $Y_{1,3}=0.5862$ since, theoretically, this would happen with probability 0). FLGI, Forward‐Looking Gittins Index

Figure 1 illustrates how the FLGI probabilities for block two, given the data in block one, are computed via a probability tree. Given that the experimental treatment has the unique maximum Gittins index, the first patient of the second block is allocated to the experimental treatment with probability 1. When the second patient of the second block is to be allocated, we need to have observed the (random) outcome of the first patient in this block, denoted by $Y_{1,3}$, in order to update the indices and determine the optimal action. The updated prior parameters for the experimental treatment, as a function of the observed information on this treatment and given the previous optimal action, are: ${Ỹ}_{1,3}=Y_{1,3}/(n_{1,3}+2),{\tilde{S}}_{1,3}={\left(\left(1/2)+(Y_{1,3}^{2}/(n_{1,3}+2)\right)\right)}^{1∕2}$, and $n_{1,3}=1$. Thus, the index for the experimental treatment can be expressed as a function of the random outcome from patient three as follows: $G_{1}\left({Ỹ}_{1,3},{\tilde{S}}_{1,3},n_{1,3}=1\right)=(Y_{1,3}/3)+{\left(\left(1/2)+(Y_{1,3}^{2}/3\right)\right)}^{1∕2}G_{1}\left(0,1,3,0.995\right),$ with $G_{1}\left(0,1,3,0.995\right)=4.6049$.

For the control treatment, we have no new information and so its index remains unchanged at $G_{0}\left({Ỹ}_{0,3},{\tilde{S}}_{0,3},n_{0,3}\right)=3.805$. According to the Gittins index rule, it is optimal to allocate the control treatment to the second patient in the block if and only if $G_{1}\left({Ỹ}_{1,3},{\tilde{S}}_{1,3},n_{1,3}\right)<G_{0}\left({Ỹ}_{0,3},{\tilde{S}}_{0,3},n_{0,3}\right)$, which happens when $-0.9508<Y_{1,3}<0.5862$. Since $Y_{1,3}$ is a standard normal random variable, this happens with probability 0.5503, that is, $Pr\left(Y_{1,3}\leq 0.5862\right)-Pr\left(Y_{1,3}\leq -0.9508\right)=0.5503$. If $Y_{1,3}<-0.9508$ or $Y_{1,3}>0.5862$, which happens with probability 0.4497, then $G_{1}\left({Ỹ}_{1,3},{\tilde{S}}_{1,3},n_{1,3}\right)>G_{0}\left({Ỹ}_{0,3},{\tilde{S}}_{0,3},n_{0,3}\right)$ and the second patient in the second block is optimally allocated to the experimental treatment. Notice that if $Y_{1,3}=-0.9508$ or $Y_{1,3}=0.5862$, the index values are equal and it is optimal to allocate any of the two treatments. In theory, this would happen with probability 0 since $Y_{k,t}$ is a continuous variable. However, in practice, if this were to happen, we would randomize with probability 0.5. Hence, the normal FLGI procedure would randomize both patients in this block to receive the experimental treatment with probability ((1+(1×0.4497))/2)=0.7249, and the control treatment with probability ((0+(1×0.5503))/2)=0.2751. Continuing this example for larger block sizes using Monte Carlo simulation, the allocation probabilities to the experimental and control arm, respectively, are (0.6565,0.3435) for b=3,(0.5151,0.4849) for b=4,(0.4370,0.5630) for b=5, and (0.3051,0.6949) for b=10.

## 3 SIMULATION STUDY

### 3.1 Alternative designs and performance measures

Next, we will report simulations that compare the FLGI for a normally distributed endpoint against the following existing randomization procedures:

(1) *Equal Randomization (ER)*, where each patient is randomly allocated to one of the K+1 arms with equal probability, 1∕(K+1). ER is predominant in practice (implemented, eg, by a permuted‐block randomization), thus it will be used as a reference to compare all designs.
(2) *Modified Zhang and Rosenberger (MZR)*, introduced by Zhang and Rosenberger (2006) and later modified by Biswas and Bhattacharya (2009) to allow for negative mean responses. The rule aims at minimizing the total of inverse mean responses, that is, $n_{0,T}∕\mu_{0}+n_{1,T}∕\mu_{1}$. This design results in the following optimal allocation proportion $\rho^{*}$
  $$\rho *=\left\{ \begin{array}{cc} c & if\;\left\{\mu_{0},\mu_{1}>0\;\text{and}\;\rho_{c}<c\right\}\;or\;\left\{\mu_{0},\mu_{1}<0,\;\frac{\sigma_{0}}{\sigma_{1}}>\sqrt{\frac{\mu_{1}}{\mu_{0}}}\right\}\;or\;\left\{\mu_{0}<0,\;\mu_{1}>0\right\},\\ \rho_{c} & if\;\left\{\mu_{0},\mu_{1}>0,\;c\leq \rho_{c}\leq 1-c\right\},\\ 1-c & if\;\{\mu_{0},\mu_{1}>0,\;\rho_{c}>1-c\}\;or\;\left\{\mu_{0},\mu_{1}<0,\;\frac{\sigma_{0}}{\sigma_{1}}<\sqrt{\frac{\mu_{1}}{\mu_{0}}}\right\}\;or\;\left\{\mu_{0}>0,\;\mu_{1}<0\right\}, \end{array}\right.$$
  where $\rho_{c}=\sigma_{0}\sqrt{\mu_{0}}\;∕\left(\sigma_{0}\sqrt{\mu_{0}}+\sigma_{1}\sqrt{\mu_{1}}\right)$ and c∈[0,1∕2]. The initial parameter estimates are obtained by allocating the first $n^{ER}$ patients using ER. After that, estimates of the unknown parameters $\mu_{k}$ and $\sigma_{k}$ are sequentially updated based on the current data available.
(3) *Constrained Gittins Index (GI) Rule* is a procedure based on Gittins indices proposed by Wang (1991a) and further studied by Coad (1991b). However, unlike the FLGI, Constrained GI is not implemented in terms of probabilities, and hence is not strictly randomized. This is a practical limitation and explains why Constrained GI has been neglected as a comparator within the RAR literature. The rule is defined as follows: if $n_{0,t}^{c}<n_{1,t}$, allocate the next patient to arm 0; if $n_{1,t}^{c}<n_{0,t}$, allocate the next patient to arm 1; else, allocate the next patient to the treatment with the largest Gittins index (randomizing if they are equal). The parameter c≥1 is a *tuning* parameter; c=1 corresponds to ER, and the Gittins index is eventually recovered as c→∞. Following Wang (1991a), we fix c=2 in our simulations.
(4) *Thompson Sampling (TS)* randomizes patients to arms based on their posterior probability of being the “best” arm. Specifically, we consider a version of Thompson (1933) suggested by Thall and Wathen (2007), where the probability of allocating treatment k to patients in block j is computed as $Pr{\left(\max_{i}\mu_{i}=\mu_{k}|{\tilde{x}}_{\left(j-1\right)b}\right)}^{c}∕\sum_{k=0}^{K}Pr{\left(\max_{i}\mu_{i}=\mu_{k}|{\tilde{x}}_{\left(j-1\right)b}\right)}^{c}$, where ${\tilde{x}}_{t}=\left({\tilde{y}}_{0,t},{\tilde{s}}_{0,t},n_{0,t},\text{…},{\tilde{y}}_{K,t},{\tilde{s}}_{K,t},n_{K,t}\right)$ and c=(j−1)b∕2T is a *tuning* parameter that recovers ER when c=0 and TS when c=1.
(5) Trippa *et al*. (2012) *Procedure (TP)* randomizes patients similarly to TS, but also protects allocation to the control arm. We have implemented TP as in Villar *et al*. (2015b).
(6) *Controlled FLGI (CFLGI)* is a variant of the FLGI design proposed in Villar *et al*. (2015b) which, similarly to TP, protects allocation to the control arm by ensuring that the corresponding allocation probability is always at least 1∕(K+1).
(7) Gwise *et al*. (2011) propose a design for comparing K+1 arms with heteroscedasticity. After an initial ER phase, patient t+1 is allocated to arm k with probability $\left({\hat{\sigma }}_{k,t}^{2}∕n_{k,t})/({\hat{\sigma }}_{0,t}^{2}∕n_{0,t}+\text{⋯}+{\hat{\sigma }}_{K,t}^{2}∕n_{K,t}\right)$, where ${\hat{\sigma }}_{k,t}^{2}$ is the estimated sample variance of the first t responses on arm k.

Note that MZR and Constrained GI are fully sequential and only defined for the two‐armed case, while TP and CFLGI apply only to the multi‐armed case. For all of the rules which require specification of a joint prior distribution on $\mu_{k}$ and $\sigma_{k}^{2}$, we take the same approach as with the FLGI. For the index based designs, a discount factor of d=0.995 is used, and the allocation probabilities defined in the FLGI designs, TS and TP, are computed using a Monte Carlo approximation based on 100 replicates. Additionally, we implement the doubly adaptive biased coin design by Hu and Zhang (2004) with the target allocation proportions taken to be the corresponding FLGI probabilities for b=T under $H_{0}$ and $H_{1}$.

To evaluate the performance of all designs, we consider patient benefit and usual inferential measures. The former includes: (a) the expected proportion of patients in the trial allocated to the superior treatment, $E\left(p^{*}\right)$, and (b) the percentage change in expected total outcome (ETO) for rule r relative to the theoretical expected total outcome for ER $\left(ETO_{ER}\right)$, computed as $100\times \left(ETO_{r}-ETO_{ER}\right)∕ETO_{ER}$ and denoted in Tables 1 and 2 by *RelETO*%. For the inferential measures, we focus on standard operating characteristics, including: power, 1−β; type I error rate, α; and bias in the maximum likelihood estimate of the treatment effect, $E\left(\hat{\Delta }-\Delta \right),$ with $\Delta =\mu_{k}-\mu_{0}$ and $\hat{\Delta }=\left({\hat{\mu }}_{k}-{\hat{\mu }}_{0}\right)$. For the multi‐armed case, we report both the marginal power (ie, power to reject $H_{0,k^{*}}$, where $k^{*}$ is the best arm) and the bias for the best experimental arm under $H_{1}$. Note that under $H_{0}$, we take $k^{*}$ to be the control arm.

**Table 1:** Comparison of performance measures for a two‐armed trial using different designs when the variance is assumed unknown (with the exception of FLGI‐known) and T=72, averaged over 50 000 trial replications

	$\mu_{0}=\mu_{1}=0.155$					$\mu_{0}=0.155,\;\mu_{1}=0.529$			
Design	$t_{1-\alpha }$	α	$E\left(p^{*}\right)$ (SD)	*Rel* ETO% (SD)	Bias (SD)	1−β	$E\left(p^{*}\right)$ (SD)	*Rel* ETO% (SD)	Bias (SD)
ER
*b *= 1	1.654	0.0518	0.49990 (0.06)	–0.19 (5.45)	–0.0005 (0.15)	0.7884	0.5005 (0.06)	0.20 (5.66)	–0.0013 (0.15)
FLGI‐known									
b=1	1.991	0.0526	0.4997 (0.33)	–0.06 (5.42)	–0.0004 (0.49)	0.2290	0.8823 (0.16)	41.69 (7.01)	0.2167 (0.45)
b=2	1.969	0.0505	0.5002 (0.32)	0.34 (5.42)	–0.0003 (0.44)	0.2701	0.8777 (0.16)	41.27 (6.85)	0.1870 (0.41)
b=6	1.911	0.0481	0.4987 (0.29)	–0.03 (5.45)	0.0010 (0.34)	0.3599	0.8605 (0.14)	39.38 (6.59)	0.1147 (0.30)
b=9	1.864	0.0492	0.4983 (0.28)	–0.15 (5.43)	0.0008 (0.30)	0.4235	0.8483 (0.13)	38.14 (6.53)	0.0843 (0.26)
b=18	1.766	0.0513	0.5017 (0.24)	0.14 (5.44)	–0.0010 (0.23)	0.5653	0.8074 (0.12)	33.72 (6.30)	0.0389 (0.20)
b=36	1.682	0.0495	0.5000 (0.19)	0.26 (5.45)	0.0001 (0.17)	0.7124	0.7139 (0.09)	23.25 (5.98)	0.0087 (0.17)
FLGI									
b=1	2.1820	0.0525	0.5013 (0.29)	–0.15 (5.43)	0.0013 (0.29)	0.3289	0.8712 (0.12)	40.62 (6.39)	0.0955 (0.27)
b=2	2.1590	0.0497	0.5016 (0.28)	0.21 (5.45)	0.0014 (0.28)	0.3432	0.8651 (0.12)	39.95 (6.41)	0.0902 (0.26)
b=6	2.1180	0.0477	0.4985 (0.27)	0.18 (5.42)	–0.0008 (0.26)	0.3790	0.8521 (0.12)	38.48 (6.36)	0.0801 (0.25)
b=9	2.0450	0.0514	0.5011 (0.26)	–0.01 (5.41)	0.0019 (0.25)	0.4236	0.8412 (0.12)	37.13 (6.36)	0.0698 (0.24)
b=18	1.8980	0.0517	0.5008 (0.24)	–0.04 (5.42)	0.0005 (0.22)	0.5277	0.8047 (0.12)	33.30 (6.23)	0.0356 (0.20)
b=36	1.7330	0.0505	0.4997 (0.18)	0.01 (5.43)	–0.0009 (0.18)	0.6973	0.7128 (0.09)	23.23 (6.00)	0.0097 (0.17)
FLGI‐HZ (γ=2)									
b=1	1.658	0.0509	0.5000 (0.05)	0.12 (5.43)	0.0008 (0.15)	0.6510	0.7784 (0.04)	30.46 (5.51)	0.0001 (0.18)
b=2	1.660	0.0509	0.5003 (0.05)	–0.14 (5.44)	0.0004 (0.15)	0.6499	0.7786 (0.04)	30.61 (5.54)	–0.0007 (0.18)
b=6	1.688	0.0487	0.5001 (0.05)	0.16 (5.42)	0.0000 (0.15)	0.6417	0.7781 (0.04)	30.40 (5.54)	–0.0001 (0.18)
b=9	1.661	0.0529	0.4994 (0.05)	0.43 (5.43)	0.0017 (0.15)	0.6501	0.7777 (0.04)	30.27 (5.52)	–0.0004 (0.18)
b=18	1.684	0.0490	0.4999 (0.05)	0.11 (5.41)	0.0003 (0.15)	0.6570	0.7644 (0.04)	28.99 (5.54)	–0.0011 (0.18)
b=36	1.665	0.0516	0.5000 (0.05)	0.10 (5.41)	0.0017 (0.15)	0.7779	0.5865 (0.05)	9.57 (5.64)	0.0003 (0.15)
TS									
b=1	1.751	0.0496	0.4999 (0.11)	–0.1108 (5.44)	–0.0001 (0.17)	0.7425	0.6961 (0.11)	21.35 (6.14)	0.0302 (0.19)
b=2	1.739	0.0497	0.4997 (0.11)	–0.0431 (5.41)	–0.0016 (0.17)	0.7479	0.6934 (0.11)	21.27 (6.11)	0.0290 (0.19)
b=6	1.741	0.0513	0.4994 (0.11)	0.3098 (5.44)	–0.0001 (0.17)	0.7489	0.6825 (0.10)	19.88 (6.14)	0.0257 (0.18)
b=9	1.729	0.0499	0.5000 (0.10)	0.1311 (5.42)	0.0001 (0.17)	0.7547	0.6747 (0.10)	18.95 (6.13)	0.0229 (0.18)
b=18	1.722	0.0494	0.5008 (0.10)	0.4446 (5.42)	0.0013 (0.16)	0.7602	0.6509 (0.10)	16.40 (6.11)	0.0184 (0.17)
b=36	1.697	0.0507	0.4999 (0.08)	0.4332 (5.41)	0.0013 (0.16)	0.7726	0.6040 (0.10)	11.45 (6.07)	0.0095 (0.16)
CGI
(c = 2)	1.887	0.0496	0.4871 (0.28)	0.19 (5.42)	0.0004 (0.24)	0.4298	0.8294 (0.11)	37.59 (6.19)	0.0340 (0.21)
MZR									
$n^{\text{ER}}=2$	1.794	0.0516	0.5005 (0.19)	0.2794 (5.41)	0.0002 (0.19)	0.7471	0.6569 (0.12)	17.15 (5.76)	0.0229 (0.17)
$n^{\text{ER}}=6$	1.780	0.0507	0.4998 (0.17)	0.3487 (5.43)	0.0001 (0.18)	0.7632	0.6414 (0.10)	15.47 (5.49)	0.0202 (0.16)
$n^{\text{ER}}=11$	1.751	0.0508	0.5001 (0.14)	–0.2534 (5.41)	0.0007 (0.17)	0.7755	0.6173 (0.08)	12.86 (5.29)	0.0155 (0.16)
Gwise									
$n^{\text{ER}}=2$	1.877	0.0495	0.4997 (0.13)	–0.08 (5.43)	0.0012 (0.18)	0.7193	0.4999 (0.13)	–0.11 (6.47)	–0.0013 (0.18)
$n^{\text{ER}}=6$	1.697	0.0482	0.5003 (0.06)	–0.08 (5.45)	–0.0000 (0.15)	0.7833	0.5005 (0.06)	0.02 (5.67)	–0.0013 (0.15)
$n^{\text{ER}}=11$	1.705	0.0492	0.4999 (0.06)	0.07 (5.41)	0.0007 (0.15)	0.7837	0.5000 (0.06)	–0.20 (5.66)	0.0000 (0.15)

*Note:* The true variance of the response is $\sigma_{k}^{2}=0.64^{2}$ for k∈{0,1}.

Abbreviations: ER, Equal Randomization; ETO, Expected Total Outcome; FLGI, Forward‐Looking Gittins Index; MZR, Modified Zhang and Rosenberger; TS, Thompson Sampling; CGI, Constrained Gittins Index; FLGI‐HZ, Forward‐Looking Gittins Index Implemented Using Hu and Zhang.

**Table 2:** Comparing the performance measures of the FLGI rule when the variance is incorrectly assumed to be known (and equal to $0.64^{2}$), in at least one of the two arms, with those obtained when the variance is assumed unknown (but with an initial estimate, ${\tilde{s}}_{k,0}^{2}$, of $0.64^{2}$)

	$\mu_{0}=\mu_{1}=0.155$					$\mu_{0}=0.155,\;\mu_{1}=0.529$			
b	$t_{1-\alpha }$	α	$E\left(p^{*}\right)$ (SD)	*Rel* ETO% (SD)	Bias (SD)	1−β	$E\left(p^{*}\right)$ (SD)	*Rel* ETO% (SD)	Bias (SD)
*(i) FLGI‐known with* $\sigma_{0}^{2}=\sigma_{1}^{2}=\frac{1}{2}\times 0.64^{2}$									
1	2.001	0.0509	0.4983 (0.27)	–0.04 (3.84)	0.0013 (0.26)	0.4722	0.9215 (0.07)	46.01 (4.34)	0.1430 (0.29)
2	1.977	0.0505	0.5010 (0.26)	0.17 (3.83)	–0.0007 (0.24)	0.5422	0.9142 (0.07)	45.22 (4.32)	0.1209 (0.26)
6	1.925	0.0525	0.4986 (0.24)	0.12 (3.85)	0.0009 (0.20)	0.6642	0.8952 (0.07)	43.29 (4.33)	0.0771 (0.21)
9	1.887	0.0518	0.5002 (0.23)	0.07 (3.82)	–0.0004 (0.18)	0.7241	0.8814 (0.07)	41.77 (4.30)	0.0545 (0.18)
18	1.798	0.0509	0.4999 (0.21)	0.14 (3.83)	0.0010 (0.15)	0.8406	0.8370 (0.07)	36.90 (4.29)	0.0224 (0.14)
36	1.698	0.0500	0.5006 (0.16)	–0.03 (3.83)	–0.0005 (0.12)	0.9341	0.7331 (0.06)	25.53 (4.19)	0.0050 (0.12)
*(ii) FLGI with* $\sigma_{0}^{2}=\sigma_{1}^{2}=\frac{1}{2}\times 0.64^{2}$									
1	2.280	0.0502	0.5014 (0.30)	–0.04 (3.84)	0.0017 (0.21)	0.4301	0.9177 (0.07)	45.60 (4.33)	0.0641 (0.20)
2	2.209	0.0484	0.4987 (0.29)	0.04 (3.83)	–0.0013 (0.20)	0.4713	0.9131 (0.07)	45.15 (4.32)	0.0602 (0.20)
6	2.132	0.0491	0.4987 (0.28)	0.01 (3.81)	–0.0017 (0.19)	0.5520	0.9008 (0.07)	43.88 (4.35)	0.0537 (0.19)
9	2.080	0.0505	0.4991 (0.27)	0.04 (3.84)	–0.0007 (0.18)	0.5958	0.8894 (0.07)	42.55 (4.33)	0.0427 (0.18)
18	1.897	0.0516	0.5004 (0.24)	0.04 (3.84)	–0.0004 (0.16)	0.7604	0.8466 (0.07)	37.95 (4.29)	0.0186 (0.15)
36	1.751	0.0501	0.5000 (0.19)	–0.22 (3.83)	–0.0002 (0.12)	0.9110	0.7401 (0.06)	26.33 (4.18)	0.0035 (0.12)
*(iii) FLGI‐known with* $\sigma_{0}^{2}=\sigma_{1}^{2}=2\times 0.64^{2}$									
1	1.940	0.0505	0.4998 (0.37)	–0.04 (7.69)	0.0024 (0.81)	0.1517	0.8106 (0.27)	34.25 (10.54)	0.2656 (0.74)
2	1.924	0.0481	0.4997 (0.36)	0.13 (7.64)	–0.0015 (0.72)	0.1719	0.8116 (0.26)	34.29 (10.29)	0.2279 (0.66)
6	1.865	0.0484	0.4999 (0.33)	–0.28 (7.70)	–0.0008 (0.54)	0.2233	0.8018 (0.23)	33.06 (9.85)	0.1441 (0.48)
9	1.790	0.0529	0.4998 (0.31)	–0.21 (7.69)	–0.0009 (0.45)	0.2730	0.7935 (0.21)	32.21 (9.55)	0.1068 (0.40)
18	1.747	0.0514	0.4998 (0.27)	0.49 (7.70)	0.0006 (0.33)	0.3454	0.7593 (0.18)	28.34 (9.10)	0.0487 (0.30)
36	1.677	0.0497	0.5003 (0.20)	–0.03 (7.66)	0.0050 (0.25)	0.4532	0.6837 (0.14)	20.27 (8.53)	0.0108 (0.24)
*(iv) FLGI with* $\sigma_{0}^{2}=\sigma_{1}^{2}=2\times 0.64^{2}$									
1	2.386	0.0518	0.5013 (0.32)	–0.04 (7.69)	0.0009 (0.45)	0.1944	0.8117 (0.21)	34.37 (9.55)	0.1326 (0.41)
2	2.323	0.0501	0.4975 (0.31)	–0.21 (7.64)	–0.0037 (0.43)	0.2047	0.8088 (0.21)	33.57 (9.50)	0.1292 (0.4)
6	2.255	0.0502	0.5008 (0.29)	0.05 (7.68)	0.0000 (0.40)	0.2184	0.7945 (0.20)	32.22 (9.37)	0.1117 (0.38)
9	2.138	0.0525	0.5004 (0.28)	–0.07 (7.69)	0.0016 (0.38)	0.2413	0.7827 (0.19)	31.01 (9.27)	0.0936 (0.35)
18	1.888	0.0514	0.4974 (0.25)	–0.02 (7.63)	–0.0028 (0.32)	0.3346	0.7511 (0.17)	27.18 (8.97)	0.0517 (0.29)
36	1.753	0.0480	0.5002 (0.19)	0.13 (7.69)	–0.0012 (0.25)	0.4470	0.6748 (0.13)	19.11 (8.48)	0.0145 (0.24)
*Heterogeneous variances*									
*(v) FLGI‐known with* $\sigma_{0}^{2}=0.64^{2},\;\sigma_{1}^{2}=\frac{1}{2}\times 0.64^{2}$									
1	1.9900	0.0502	0.4284 (0.30)	0.03 (4.61)	0.1030 (0.38)	0.1828	0.9203 (0.09)	45.84 (4.32)	0.2522 (0.41)
2	1.9710	0.0487	0.4358 (0.29)	–0.35 (4.60)	0.0902 (0.35)	0.2200	0.9102 (0.09)	44.85 (4.32)	0.2038 (0.37)
6	1.9170	0.0487	0.4587 (0.27)	0.26 (4.63)	0.0583 (0.28)	0.3399	0.8857 (0.09)	42.06 (4.35)	0.1271 (0.28)
9	1.8730	0.0508	0.4666 (0.26)	–0.08 (4.65)	0.0477 (0.24)	0.4222	0.8703 (0.09)	40.49 (4.36)	0.0952 (0.24)
18	1.7890	0.0532	0.4836 (0.23)	0.09 (4.68)	0.0280 (0.19)	0.5893	0.8240 (0.09)	35.37 (4.43)	0.0427 (0.18)
36	1.7060	0.0533	0.4992 (0.18)	0.07 (4.70)	0.0131 (0.15)	0.7697	0.7237 (0.08)	24.42 (4.56)	0.0117 (0.15)
*(vi) FLGI with* $\sigma_{0}^{2}=0.64^{2},\;\sigma_{1}^{2}=\frac{1}{2}\times 0.64^{2}$									
1	2.425	0.0489	0.4822 (0.31)	–0.07 (4.72)	0.0341 (0.26)	0.2655	0.8897 (0.11)	42.51 (4.58)	0.1104 (0.26)
2	2.331	0.0524	0.4789 (0.30)	–0.07 (4.69)	0.0323 (0.25)	0.2969	0.8833 (0.11)	41.94 (4.59)	0.1044 (0.25)
6	2.266	0.0485	0.4752 (0.28)	0.05 (4.74)	0.0282 (0.24)	0.3431	0.8674 (0.11)	40.08 (4.62)	0.0927 (0.24)
9	2.185	0.0510	0.4722 (0.28)	–0.37 (4.72)	0.0263 (0.23)	0.3861	0.8562 (0.11)	38.81 (4.62)	0.0813 (0.23)
18	1.977	0.0489	0.4721 (0.25)	0.06 (4.75)	0.0197 (0.19)	0.5261	0.8158 (0.10)	34.69 (4.60)	0.0418 (0.19)
36	1.778	0.0511	0.4786 (0.19)	–0.04 (4.72)	0.0120 (0.15)	0.7429	0.7226 (0.09)	24.28 (4.60)	0.0111 (0.15)
*(vii) FLGI‐known with* $\sigma_{0}^{2}=0.64^{2},\;\sigma_{1}^{2}=2\times 0.64^{2}$									
1	1.981	0.0489	0.5892 (0.34)	0.30 (6.45)	–0.1878 (0.65)	0.2669	0.7882 (0.29)	31.44 (11.25)	0.0885 (0.64)
2	1.941	0.0524	0.5747 (0.34)	0.33 (6.51)	–0.1584 (0.59)	0.3005	0.7958 (0.27)	32.39 (10.90)	0.0819 (0.55)
6	1.855	0.0519	0.5517 (0.31)	0.01 (6.53)	–0.1035 (0.45)	0.3741	0.8034 (0.23)	33.30 (10.18)	0.0586 (0.40)
9	1.821	0.0482	0.5422 (0.30)	–0.03 (6.56)	–0.0806 (0.38)	0.4119	0.7995 (0.21)	32.71 (9.84)	0.0454 (0.33)
18	1.735	0.0489	0.5194 (0.26)	–0.34 (6.59)	–0.0469 (0.28)	0.5105	0.7758 (0.17)	30.16 (9.15)	0.0197 (0.24)
36	1.665	0.0475	0.5007 (0.20)	–0.04 (6.63)	–0.0225 (0.22)	0.6098	0.6982 (0.12)	21.74 (8.20)	0.0004 (0.20)
*(viii) FLGI with* $\sigma_{0}^{2}=0.64^{2},\;\sigma_{1}^{2}=2\times 0.64^{2}$									
1	2.238	0.0517	0.5204 (0.32)	–0.05 (6.70)	–0.0480 (0.38)	0.2806	0.8522 (0.19)	38.78 (9.50)	0.0774 (0.34)
2	2.207	0.0484	0.5249 (0.31)	0.24 (6.65)	–0.0433 (0.37)	0.2885	0.8507 (0.18)	38.63 (9.40)	0.0710 (0.32)
6	2.095	0.0509	0.5290 (0.29)	–0.06 (6.69)	–0.0377 (0.34)	0.3353	0.8405 (0.17)	37.31 (9.28)	0.0637 (0.30)
9	2.039	0.0491	0.5297 (0.28)	–0.07 (6.71)	–0.0367 (0.33)	0.3554	0.8299 (0.16)	36.31 (9.14)	0.0501 (0.28)
18	1.839	0.0497	0.5284 (0.25)	0.05 (6.72)	–0.0296 (0.27)	0.4708	0.7943 (0.14)	32.01 (8.71)	0.0220 (0.23)
36	1.700	0.0489	0.5222 (0.19)	0.15 (6.70)	–0.0182 (0.21)	0.6050	0.7017 (0.11)	21.95 (8.05)	0.0030 (0.20)

*Note:* In the upper half of the table, the true variance of the response is actually half or double 0.64^2^ (as shown) and in the lower half, the true variances are heterogeneous. These results are averaged over 50 000 replications for a trial of size *T* = 72.

Abbreviation: FLGI, Forward‐Looking Gittins Index.

We consider the following hypotheses: $H_{0}:\mu_{0}=\mu_{k}\;\forall \;k$ versus the one‐sided alternatives, $H_{1,k}:\mu_{0}<\mu_{k}$ for some k>0 considered the best arm. We will use the test statistic $T_{k}=\left({\bar{Y}}_{k}-{\bar{Y}}_{0}\right)∕\sqrt{\left({\hat{\sigma }}_{k}^{2}/n_{k,T})+({\hat{\sigma }}_{0}^{2}/n_{0,T}\right)}\;\text{for}\;k=1,\text{…},K,$ where ${\bar{Y}}_{k}$ and ${\hat{\sigma }}_{k}^{2}$ are the sample mean and sample variance, respectively, of arm k at the end of the trial. In the multi‐armed case, we consider the joint distribution of $T_{1},\text{…},T_{K}$ and use a critical value, $t_{1-\alpha }$, to achieve a family‐wise type I error rate (FWER) close to the specified α, where FWER is defined as the probability of obtaining at least one false positive within the family of hypotheses.

### 3.2 A two‐armed trial

To motivate this scenario, we use the example in Karrison *et al.* (2007) of a two‐armed phase II cancer trial, in which the primary endpoint is the ratio of tumor size at the time of follow‐up to that at baseline for patient *t* under treatment *k*, that is, the change in tumor size, denoted by *C_k,t_*. After a log‐transformation, *C_k,t_* is continuous and approximately normally distributed, as shown by Lavin (1981). In keeping with our assumption that a larger outcome is desirable, we add a minus sign to re‐express the endpoint as a measure of tumor reduction. Under the assumption that *Y*_0,*t*_ = –log(*C*_0,*t*_) ∼ *N*(0.155, 0.64^2^) and *Y*_1,*t*_ = –log(*C*_1,*t*_) ∼ *N*(0.529, 0.64^2^), the total sample size required to detect this treatment difference with approximately 80% power at the *α* = .05 significance level and assuming complete observations is *T *= 72.

#### 3.2.1 Results

Table 1 displays the results from 50 000 replications of the trial when we assume unknown variance. As expected, under $H_{0}$ all the designs are equal in terms of patient benefit (*Rel* ETO%≈0 and $E\left(p^{*}\right)\approx 0.50$). The main difference between designs under the null is the variability of the allocations, represented by the standard deviations (SD) of $p^{*}$, with ER and FLGI (for b=1) being the least and most variable, respectively. As the block size increases, changes in the allocation probabilities are based on more data and the FLGI becomes less variable. The index based procedures tend to be more variable because they aim at maximizing patient response. For example, the Constrained GI design also has a large variability which is comparable to that of the FLGI. For the MZR design, the variability of the allocations decreases as the size of the initial ER period, $n^{ER}$, increases. The variability of the FLGI is also markedly reduced when implemented using Hu and Zhang (2004), labeled as FLGI‐HZ in Table 1. In terms of the bias of the treatment effect estimator, all are (on average) unbiased under $H_{0}$. Note that we have used adjusted t‐critical values to control type I error rates for all designs following the approach used in Smith and Villar (2018). The (unreported) type I error inflation incurred for the FLGI when using the usual $t_{0.95}$ critical value is approximately 11% for b=1 and it decreases as the block size grows, as expected. A similar level and pattern of inflation occurs for TS.

**Figure 2:**
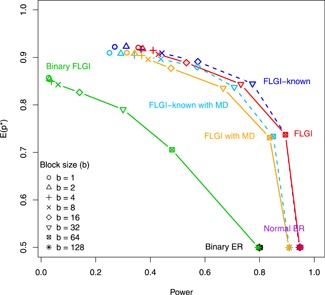
The trade‐off between the expected proportion of patients allocated to the superior arm, $E\left(p^{*}\right)$, and power for the: binary ER, normal ER, binary FLGI, normal FLGI, and normal FLGI with missing data (MD) imputed in an online fashion for block sizes b=(1,2,4,8,16,32,64,128) in a two‐armed trial of size T=128. The latter two designs are shown when assuming both an unknown variance and known (correct) variance (dashed line and labeled as FLGI‐known). ER, equal randomization; FLGI, Forward‐Looking Gittins Index [This figure appears in color in the electronic version of this article, and any mention of color refers to that version]

The results under $H_{1}$, in which we are testing for superiority of arm 1 (the experimental arm), show more contrasts among designs. First, we focus on the FLGI design and the effect of varying the block size on the power versus patient benefit trade‐off. When b=1, the FLGI design is statistically identical to the fully sequential Gittins index rule and so favors patient response. At the other extreme, when b=T, the FLGI design is equivalent to ER and therefore favors power. Thus, consistent with the findings for the binary case, Table 1 shows that as b increases under $H_{1}$, the patient benefit measures (and corresponding standard deviations) decrease, while the power increases (at a faster rate) which illustrates the natural tension between these two conflicting goals. This relationship is depicted visually in Figure 2 for T=128.

In terms of the patient benefit measures, the index based designs (namely the FLGI and Constrained GI) perform the best out of all the designs considered. Relative to ER, for a moderate block size of b=9, the FLGI allocates approximately 34% more patients to the superior treatment (equivalent to 25 patients). Moreover, the expected total tumor size reduction is just over 37% greater than that obtained when using ER. Even for a large block size of b=36, the FLGI allocates approximately 21% more patients to arm 1 and achieves an expected total tumor size reduction 23% larger than ER. All other block sizes for the FLGI have a total tumor size reduction at least 30% greater than ER, on average. The Constrained GI is shown to perform similarly to the FLGI when b=9. TS has a total tumor size reduction rate of at least 20% relative to ER for small b, on average, whereas MZR falls below this for all $n^{ER}$.

As mentioned above, the cost of these patient benefit gains is a severe reduction in the power compared to that of ER. However, this is ameliorated as b increases or by implementing the FLGI probabilities using Hu and Zhang (2004). The ER design attains an unbiased treatment effect estimator, as expected, with the largest relative bias exhibited by the FLGI design when b=1 (ie, the GI design). This makes sense because this is the design with the biggest imbalance in favor of arm 1. As a result, ${\hat{\mu }}_{0}$ will be substantially underestimated giving rise to an overestimated $\hat{\Delta }$ (and positive bias of treatment effect). As b increases, and consequently the number of observations on arm 0 increases, the bias (and associated standard deviations) of the treatment effect estimator decreases.

These results emphasize the very important point that, in a two‐armed setting, none of the designs are uniformly better than the others for *every* performance measure since each design is tailored toward a different competing objective. This makes direct comparisons between such designs infeasible and motivates our main interest in the multi‐armed case.

Table 1 also shows the results attained by the FLGI rule when assuming the correct variance in both arms (see FLGI‐known). As expected, FLGI‐known marginally outperforms the FLGI with unknown variance in terms of patient benefit (and reduces the power) due to the additional uncertainty present in the latter. However, in practice, this is unrealistic since the true variance of the outcome is seldom known at the start of a trial. Therefore, in Table 2, we illustrate the effect of assuming an *incorrect* variance (on one, or both, of the arms) on the performance of the FLGI relative to when assuming an unknown variance. Although misspecifying the variance does not always have a negative impact on the results, and the performance may be comparable to that when assuming an unknown variance (as in Scenarios (i)–(iv)), it is important to be aware that it can sometimes lead to a considerable loss in patient benefit. This is evident in Scenario (vii) of Table 2, for example, where 6.4% fewer patients are allocated to the superior treatment (for *b* = 1) as a consequence of underestimating $\sigma_{1}^{2}$. As such, the robustness and flexibility attained by the FLGI with unknown variance makes this design more suited to practice.

### 3.3 A multi‐armed trial

We now use the phase II cancer trial setting described in Karrison *et al*. (2007) as a case study. The primary endpoint is again the change in tumor size from baseline to eight weeks. Patients were randomly assigned to one of three treatment arms: 150 mg of erlotinib plus placebo; 150 mg of erlotinib plus 200 mg of sorafenib; or 150 mg of erlotinib plus 400 mg of sorafenib. We will refer to these as the control, low dose and high dose, respectively.

Based on data from previous trials, the log ratio of tumor sizes is assumed to have a mean of 0.05 for the control (k=0), −0.07 for the low dose (k=1) and −0.13 for the high dose (k=2), with a common standard deviation of 0.346. To be consistent with our earlier assumption that larger responses are desirable, we instead consider tumor reduction. Therefore, we assume that $Y_{0,t}∼N(-0.05,0.346^{2}),\;Y_{1,t}∼N(0.07,0.346^{2})$, and $Y_{2,t}∼N(0.13,0.346^{2})$. We simulate a trial of size T=120, which should have at least 80% power using a one‐sided test at α=.10 when no correction for multiplicity is considered. In our simulations, we will ensure a one‐sided test at the α=.10 FWER level, and since we adjust for multiplicity, the power will fall slightly below 80%, illustrating the effect of correcting for multiplicity on power.

#### 3.3.1 Results

**Figure 3:**
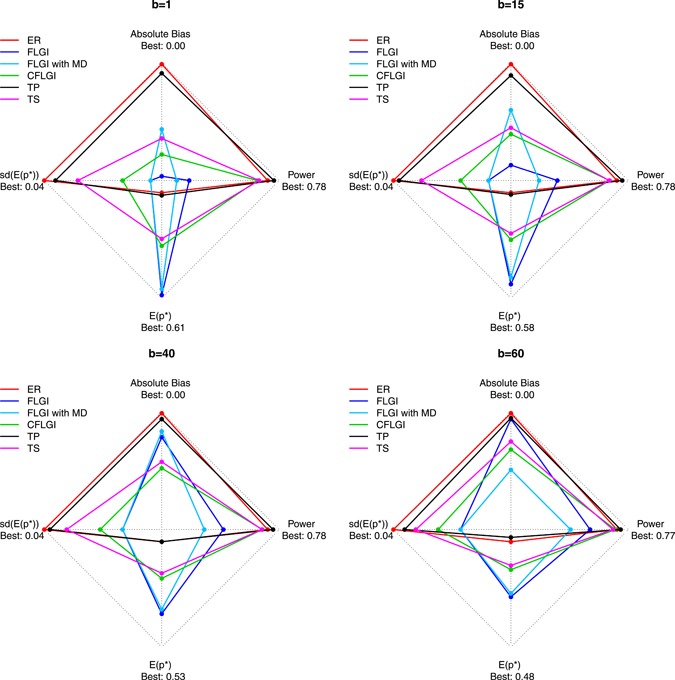
The trade‐offs between the expected proportion of patients allocated to the superior arm, $E\left(p^{*}\right)$, power, average absolute bias in the treatment effect estimate and variability of patient allocations for the different designs, including normal FLGI and normal FLGI with missing data (MD), for block sizes b=(1,15,40,60) in a three‐armed trial of size T=120 (assuming unknown variance). CFLGI, Controlled FLGI; ER, Equal Randomization; FLGI, Forward‐Looking Gittins Index; TP, Trippa Procedure; TS, Thompson Sampling [This figure appears in color in the electronic version of this article, and any mention of color refers to that version]

Under the null, the only relevant difference among designs is the variability of resulting allocations, with the rules performing the best in terms of patient benefit being the most variable. Results under the alternative hypothesis are illustrated in Figure 3 and provided in full (for both $H_{0}$ and $H_{1}$) in Table S4. Figure 3 shows a star plot summarizing the key features of each design (for blocks 1, 15, 40, and 60) where the most desirable values lie toward the *outer* edge of the star plot with the least favorable values toward the center. We see that ER performs very well with respect to power, average bias and variability, but poorly with respect to patient benefit for all block sizes, while in contrast the FLGI design performs poorly with respect to power, average bias and variability but the best with respect to patient benefit. The CFLGI and TS design have values lying near to the outer edge of the star plot for all measures, thus showing that they perform well with respect to all of the performance measures. Although CFLGI and TS have similar performances, they are not directly comparable as they attain different compromises between the competing objectives. Rather than having a flat probability protection for the control arm during the trial, the definition of the CFLGI rule could be adjusted in a similar way to TS and TP, which we expect would result in an advantage over TS in terms of patient benefit, especially for smaller trials with several arms.

## 4 DICHOTOMIZATION: PATIENT BENEFIT AND EFFICIENCY COST

Phase II cancer trials, such as the ones considered above, are traditionally conducted as single arm studies using a binary response rate as the primary endpoint, which is formed by splitting the underlying continuous data (change in tumor size) into two groups (success or failure of a treatment), that is, dichotomizing. This dichotomization is often based on the Response Evaluation Criteria in Solid Tumors (Eisenhauer *et al*., 2009) which categorizes the change in tumor size and number of lesions into four levels: complete response, partial response, stable disease, and progressive disease. A treatment is considered a success if patients experience either a partial or complete response (ie, at least a 30% reduction in the total diameter of target lesions), and a failure otherwise. If new lesions appear, or non‐target lesions grow beyond a certain percentage, this is also classed as a treatment failure.

Dichotomizing continuous data is a widely adopted approach in clinical research. However, this comes at the cost of losing power as well as raising issues such as where exactly the dichotomization cutpoint should be. Within the literature, there is a strong focus on the loss of efficiency associated with dichotomizing a continuous variable, but no mention of the cost to patients in the trial. Therefore, we will use the same two‐armed example as in Section 3.2 to compare the performance, in terms of patient benefit measures, of the continuous FLGI to the binary FLGI, as proposed in Villar *et al*. (2015b). However, since the binary FLGI compares response rates, we increase the total sample size from T=72 to 128, as this is the size required to detect an improvement from 20% to 40% with 80% power using a one‐sided test at the α=.05 level; a 77% increase on that required for the continuous case.

Figure 2 shows the efficiency costs of dichotomizing a continuous endpoint. A trial of size 128 achieves almost 100% power to detect the target treatment difference when using a continuous endpoint, as opposed to 80% power when using a binary one. Moreover, Figure 2 also illustrates that there is an important patient benefit cost of using a binary endpoint instead of a continuous one when using RAR. In particular, the normal FLGI (all versions) has not only a higher power level, but also a considerably higher expected proportion of patients on the best arm for every block size in a trial of size 128.

## 5 IMPUTING COMPLETE RESPONSES AND DROPOUTS

The patient benefit cost associated with dichotomizing requires an important practical consideration to be taken into account when interpreting it. To implement any response‐adaptive design in practice, particularly in cancer trials like those used in this paper, we need an online imputation method to account for patients who (a) die or dropout of the trial before the follow‐up time, or (b) have a complete response (since this causes the log ratio to be undefined). Two approaches have been proposed to impute these cases in Karrison *et al*. (2007) and Jaki *et al*. (2013), a review of which is provided by Wason and Jaki (2016).

So far, we have assumed that *all* patients generate an observable response, which is clearly not realistic. Whereas deaths/dropouts and complete responses are easily imputed in the binary case, there is no obvious way of translating these outcomes into continuous variables. Building upon the solution in Karrison *et al*. (2007), where the best and worst possible outcomes are used to impute complete responses and deaths/dropouts, respectively, we instead randomize from the upper tail of the (theoretical) distribution under $H_{1}$ if we observe a complete response, and from the lower tail of the null distribution to account for deaths or dropouts, regardless of which treatment the patient received. Thus, this approach allows for a response‐adaptive algorithm to be used by computing the missing values online as the trial progresses. Furthermore, choosing the missing values *randomly*, as opposed to using the same values every time, is perhaps a better reflection of reality or, at the very least, a reflection of the distributional assumptions made to determine the size of the study based on power considerations.

Figure 2 shows the results for the normal FLGI when we implement our online imputation method assuming that we observe a 4% rate of deaths or dropouts and a 1% rate of complete responses. This is illustrated under the assumption of both a known and unknown variance, labeled as FLGI‐known with missing data (MD) and FLGI with MD, respectively. These rates are consistent with values reported in Karrison *et al.* (2007). Figure 2 shows that, as expected, this missing data assumption decreases both the efficiency and patient benefit advantages, relative to the FLGI with complete observations, for both the known and unknown variance cases. Nevertheless, the imputed continuous FLGI procedure continues to greatly outperform the binary FLGI with respect to both criteria. Figure 3 suggests that similar conclusions also apply for the multi‐armed missing data case (see FLGI with MD).

## 6 DISCUSSION

The RAR literature contains relatively few procedures for a continuous endpoint assumed to be normally distributed with unknown variance, fewer still that are defined for the multi‐armed case and none that are forward‐looking. We propose the first forward‐looking RAR algorithm applicable to this case which is oriented toward an optimality criterion with respect to patient benefit.

In this paper, we have shown that using a continuous endpoint instead of dichotomizing can offer efficiency, but also patient benefit advantages, when combined with RAR. Implementing a RAR procedure, such as the FLGI, in the context of phase II cancer trials requires dealing with missing data from patients in an online fashion. The naïve imputation method suggested in this work, based on the method by Karrison *et al*. (2007), shows that there are still important benefits even if a low rate of missing observations is anticipated. Further work is needed to develop imputation methods that can be used in combination with RAR.

An important advantage of our proposed method is that it can be implemented without assuming a fixed, known, and common variance. In fact, the FLGI with unknown variance can learn about the variance simultaneously as it learns about the treatment means, and update the randomization probabilities accordingly. Additionally, the method can incorporate covariates in the way suggested by Villar and Rosenberger (2018).

The motivation of our algorithm is in the setting of clinical trials, but it applies to sequential allocation problems more generally. Future research could consider the issue of estimation following the sequential tests used in these novel designs, similar to work in Coad (1991a; 1994).

## Supporting information

Supplementary Information

Supplementary Information

Supplementary Information
