# Supplementary material for: A response‐adaptive randomization procedure for multi‐armed clinical trials with normally distributed outcomes
Source: Biometrics. 2019 Sep 19;76(1):197–209. doi: 10.1111/biom.13119 (PMC7078926; doi:10.1111/biom.13119)
Supplement: Supplementary file 2 — Supplementary Information [file BIOM-76-197-s002.pdf]

Supporting Information for "A Response-Adaptive Randomization Procedure for Multi-armed Clinical Trials with Normally Distributed Outcomes"

by S. Faye Williamson and Sofía S. Villar

Web Appendix A: The Multi-Armed Bandit Problem and the Forward-Looking Gittins Index Rule for Normally Distributed Endpoints

In this Web Appendix, we provide a more detailed description of the multi-armed bandit problem (MABP) for normally distributed endpoints, its solution by the Gittins index (GI) and an additional example of the Forward-Looking Gittins Index (FLGI) probabilities for normally distributed endpoints with a known variance.

The MABP in this case involves a multi-armed clinical trial that will test the effectiveness of K experimental treatments against a control treatment on a sample of T patients, with K and T fixed and known in advance. Patients are labeled by t ($t = 1, \ldots, T$) and treatments by k ($k = 0, \ldots, K$), where $k = 0$ denotes the control. The response of patient t allocated to arm k is a random variable denoted by $Y_{k,t}$ and assumed to follow a normal distribution $Y_{k,t} \sim N(\mu_k, \sigma_k^2)$. Without loss of generality, we also assume that a *larger* response is preferable and that σ_k^2 is known.

In order to derive the FLGI rule, we first need to obtain the GI for a normally distributed variable and the MABP associated with this trial design problem. For this purpose, we assume the following. (i) Each unknown parameter μ_k has a prior distribution $\pi_{k,0}$ at the start of the trial (before any observation has been made) which we take to be the normal prior $N(\mu_k^0, \frac{\sigma_k^2}{n_k^0})$. Note that the form of the prior when both μ_k and σ_k^2 are unknown is provided below. (ii) Patients enter the trial one-by-one and responses are observed immediately after treatment. We will remove these assumptions when we formulate the FLGI rule. (iii) Only one treatment can be allocated per patient and we let $a_{k,t}^r$ be a binary indicator variable denoting whether patient $t+1$ is assigned to treatment k for patient allocation rule r or not, given the

information available on all treatments. (iv) Given the conjugacy of the prior and normally distributed responses, prior distributions are converted into normal posterior distributions for each μ_k via Bayes' Theorem. After treating patient t, if $n_{k,t}$ responses from treatment k have been observed (each denoted by $y_{k,i}$ with $i = (1, \ldots, n_{k,t})$ and $n_{k,t} \leqslant t$), then the posterior distribution of μ_k at time t is $\pi_{k,t}(\mu_k | y_{k,1}, \ldots, y_{k,n_{k,t}}) \sim N\left(\frac{n_{k,t}\overline{y}_{k,t}+n_k^0\mu_k^0}{n_{k,t}+n_k^0}, \frac{\sigma_k^2}{n_{k,t}+n_k^0}\right)$ by Bayes' Theorem, where $\overline{y}_{k,t} = \frac{1}{n_{k,t}} \sum_{i=1}^{n_{k,t}} y_{k,i}$ is the sample mean and n_k^0 is the implicit sample size from the prior information (Spiegelhalter et al., 2004, p. 62). The posterior distribution, $\pi_{k,t}$, can be identified by the parameters $\tilde{y}_{k,t}$ (posterior mean) and $n_k^0+n_{k,t}$, which we subsequently refer to as the *state* (of the bandit) (Gittins et al., 2011). Note that when the variance is *unknown*, an additional parameter $\tilde{s}_{k,t}^2$, denoting the posterior variance of patient t on arm k, is required to identify $\pi_{k,t}$ and in this case, we need to specify a *joint* prior distribution for μ_k and σ_k^2 at the start of the trial. We take this to be the normal-inverse-gamma distribution (where the variance follows an inverse-gamma distribution and the mean, conditional on the variance, has a normal distribution). Consequently, the marginal prior distribution for μ_k has a Student's t-distribution. When we observe an outcome $y_{k,t+1}$ from patient $t+1$ on arm k, the state $(\tilde{y}_{k,t}, \tilde{s}_{k,t}, n_k^0 + n_{k,t})$ is updated as follows

$$\left(\frac{(n_k^0 + n_{k,t})\tilde{y}_{k,t} + y_{k,t+1}}{n_k^0 + n_{k,t} + 1}, \left(\frac{\tilde{s}_{k,t}^2(n_k^0 + n_{k,t} - 1)}{n_k^0 + n_{k,t}} + \frac{(y_{k,t+1} - \tilde{y}_{k,t})^2}{n_k^0 + n_{k,t} + 1}\right)^{\frac{1}{2}}, n_k^0 + n_{k,t} + 1\right). \quad (1)$$

The MABP is to find a patient allocation rule r that attains the maximum expected patients' response given the initial information about the treatments before the start of the trial. Mathematically, this is expressed as

$$\max_{r \in R} \mathsf{E}^r \left[\left(\sum_{t=0}^{T-1} \sum_{k=0}^{K} d^t \mathsf{E}[Y_{k,t} | \mathbf{x}_{k,t}] a_{k,t}^r \right) \middle| \tilde{\mathbf{x}}_\mathbf{0} \right], \quad (2)$$

where $\mathbf{x}_{k,t} = (\overline{y}_{k,t}, n_{k,t}, \mu_k^0, n_k^0)$, $\tilde{\mathbf{x}}_\mathbf{0} = \{\mathbf{x}_{k,0}\}_{k=0}^K$ is the initial joint state with all the prior parameters, R is the set of admissible allocation rules, $\mathsf{E}^r[\cdot]$ denotes expectation under allocation rule r, and $0 \leqslant d < 1$ is a discount factor. In MABPs, rewards are geometrically discounted so that an infinite horizon can be considered, i.e. patient t's response yields a

reward of $d^t Y_{k,t}$ for some k. In practice, a solution that depends on d, such as the GI, can be adapted to solve an undiscounted problem with a specific finite horizon, as explained in Edwards et al. (2017, Definition 6.6).

The exact solution to (2), obtained via dynamic programming, uses a backward induction algorithm which becomes computationally infeasible very quickly as T and K grow. The GI solution, first introduced by Gittins and Jones (1979), eliminates this computational infeasibility by ensuring that the optimal solution to (2) can be obtained by simply allocating every patient to the arm with the highest GI. Similarly to equation (1) in the main paper for the unknown variance case, the GIs, $\mathcal{G}(\tilde{y}_{k,t}, \sigma_k, n_{k,t})$, for the known variance case in (2) can be expressed as

$$\mathcal{G}(\tilde{y}_{k,t}, \sigma_k, n_{k,t}) = \tilde{y}_{k,t} + \sigma_k \mathcal{G}(0, 1, n_k^0 + n_{k,t}, d), \tag{3}$$

where $\mathcal{G}(0, 1, n_k^0 + n_{k,t}, d)$ denotes the GI value of a standardized bandit problem with posterior mean 0, standard deviation 1, implicit sample size n_k^0, $n_{k,t}$ observations and discount factor d (Gittins et al., 2011, Theorem 7.13). These were first computed in Jones (1975). Table 1 shows indices corresponding to the *unknown* variance case, as used in the main paper, based on those presented in Gittins et al. (2011, Table 8.3).

We implement the solution in (3) at a very low computational cost by calculating the values of $\mathcal{G}(0, 1, n_k^0 + n_{k,t}, d)$ in advance and interpolating from the tables printed in Gittins et al. (2011, pp. 261-262). Details on how to compute these indices using value iteration can be found in Gittins et al. (2011, Chapters 7 and 8). Using (3) and the GI rule, we can compute the FLGI probabilities for normally distributed endpoints (with known variance) using equation (3) in Villar et al. (2015). We now assume that instead of enrolling patients one-by-one, patients are enrolled in groups of size b over J stages, so that $J \times b = T$. Our response-adaptive rule will sequentially randomize the next b patients among the $K + 1$

treatments at stage j ($j = 1, \dots, J$) given the data up to and including block $j-1$ according to what the GI rule would do.

Example

We now illustrate the rule's implementation using an example for the case of known variances. We calculate the FLGI probabilities using the simplest possible case of a two-arm trial testing a control treatment ($k = 0$) against an experimental treatment ($k = 1$) with a block of size two ($b = 2$) and a known, common variance of $\sigma_k^2 = \sigma^2 = 1$. We assume a prior of $\mu_k \sim N(0, 1)$ so that the initial state, $(\tilde{y}_{k,0}, n_k^0)$, is $(0, 1)$ for both $k = \{0, 1\}$. Suppose further that both patients are allocated to the control treatment in the first block of the trial resulting in responses $y_{0,1} = 3.1$ and $y_{0,2} = -0.4$. The updated state after the first observation becomes $(\tilde{y}_{0,1}, n_0^0 + n_{0,1}) = (1.55, 2)$ and after the second observation becomes $(\tilde{y}_{0,2}, n_0^0 + n_{0,2}) = (\frac{0-0.4+3.1}{3}, 3) = (0.9, 3)$. Consequently, for the second block, the prior parameters for each treatment respectively are $(0.9, 3)$ and $(0, 1)$, i.e. $\mu_0 \sim N(0.9, \frac{1}{3})$ and $\mu_1 \sim N(0, 1)$.

From equation (3), setting $d = 0.995$ and using Table 8.1 in Gittins et al. (2011), the GI for the control treatment is $\mathcal{G}_0(0.9, 1, 2) = 0.9 + \frac{0.20137}{3(1-0.995)^{\frac{1}{2}}} = 1.8493$. For the experimental treatment, we only have the information available from the initial state (since no observations have yet been observed on this arm). Thus, the corresponding GI for this arm is $\mathcal{G}_1(0, 1, 0) = 0 + \frac{0.12852}{(1-0.995)^{\frac{1}{2}}} = 1.8175$.

Given that the control treatment has the maximum GI, the first patient of the second block (i.e. patient 3) is allocated to the control treatment with probability (w.p.) 1 since there is only one optimal action possible at this point. If we denote the random outcome of this patient by $Y_{0,3}$, then the updated state for the control treatment is $(\widetilde{Y}_{0,3}, n_0^0 + n_{0,3}) = \left(\frac{0-0.4+3.1+Y_{0,3}}{4}, 4\right)$. Thus, the corresponding index for the control treatment can be expressed

as a function of the random outcome from patient three as follows: $\mathcal{G}_0(\widetilde{Y}_{0,3}, 1, 3) = \frac{Y_{0,3}+2.7}{4} + \mathcal{G}_0(0, 1, 4, 0.995) = \frac{Y_{0,3}}{4} + 1.4669$.

For the experimental treatment, we have no new information and so the corresponding index remains unchanged at 1.8175. According to the GI rule, it will be optimal to allocate the control treatment to the second patient of the second block if and only if $\mathcal{G}_0(\widetilde{Y}_{0,3}, 1, 3) > \mathcal{G}_0(0, 1, 0) = 1.8175$, that is, if $Y_{0,3} > 1.4024$. Since $Y_{0,3} \sim N(0.9, 1)$, we expect this to happen w.p. $\mathcal{P}r(Y_{0,3} > 1.4024) = 0.3077$. If $Y_{0,3} < 1.4024$, which happens w.p. 0.6923, then $\mathcal{G}_0(\widetilde{Y}_{0,3}, 1, 3) < \mathcal{G}_1(0, 1, 0)$ and the second patient of the second block is optimally allocated to the experimental treatment. Notice that if $Y_{0,3} = 1.4024$, then there is a tie in the index values and it is equally optimal to allocate any of the two treatments. Although theoretically we expect this to happen w.p. 0 (since we are dealing with a continuous distribution), in practice this is possible and if it were to happen, we would simply randomize w.p. 0.5. Hence, the probability of a patient receiving either the control or experimental treatment when using the normal FLGI procedure in this block is $\frac{1+1\times\mathcal{P}r(Y_{0,3}>1.4024)}{2} = 0.6538$ and $\frac{0+1\times\mathcal{P}r(Y_{0,3}<1.4024)}{2} = 0.3462$, respectively. Figure 1 illustrates how the FLGI probabilities for block two, given the data in block one, are computed via a probability tree.

[Table 1 about here.]

[Figure 1 about here.]

Web Appendix B: Effect of Discount Factor on FLGI Performance

A practical consideration for our design is the choice of discount factor, d. We recommend choosing d to be close to that obtained when applying the formula suggested by Wang (1991), namely, $d = 1 - 1/T$, where T is the trial size. Here, we discuss the implications of *not* following this recommendation on the performance of the FLGI (with known variance) by presenting results corresponding to $d = 0,\ 0.5$ and 0.99 in Table 2. Note that the results for $d = 0.995$ (the discount factor used throughout the main paper) are shown in (i) of

Table 3. When $d = 0$, the design is analogous to a fully myopic policy which treats every patient as if they are the last one in the trial. In contrast, the closer d is to 1, the greater the influence that potential responses from future participants have on allocation decisions made earlier in the trial, that is, the more "forward looking" the design will be. Thus, we expect the patient benefit measures to increase with d (up to a limit determined by the actual population size), as illustrated in Table 2. In particular, Table 2 shows that as d increases from 0 to 0.995 for $b = 1$, $\mathbb{E}(p^*)$ increases by 0.164, which is equivalent to 11 more patients receiving the superior arm, and the relative ETO increases by 17.77%. As a result of the greater imbalance between the treatment arms for larger d, the bias of the treatment effect estimator (under $\mathcal{H}_1$) is also increased.

Interestingly, for smaller d, we observe that the patient benefit measures increase (up to around $b = 9$) followed by a decrease. This is due to an interaction between the discount factor and block size, whereby the increase in block size counteracts the myopic effect of a small d by forcing learning and consequently improving patient benefit. However, as the block size continues to grow, the effect of the design becoming more balanced supersedes the effect of the discount factor, causing the patient benefit to now reduce. Therefore, when choosing d, it is important to consider which block size will be used.

In terms of the power of the design, it increases somewhat with the size of d as illustrated by Table 2 which shows that the power exhibited for the FLGI when $d = 0$ and $b = 1$ is 0.213 compared to 0.229 when $d = 0.995$. This makes sense because increasing d from a value that is much smaller than its recommendation for a fixed T reduces the myopic nature of the rule, meaning it will explore more of the arms (thus increasing power) and make better choices (also increasing patient benefit).

The discount factor also affects the variability of the expected allocations, which decreases considerably with the value of d under both $\mathcal{H}_0$ and $\mathcal{H}_1$. For example, Table 2 shows that

under $\mathcal{H}_1$, the standard deviation (s.d.) of $\mathbb{E}(p^*)$ when $d = 0$ and $b = 1$ is 0.43, which is 2.7 times larger than the corresponding s.d. when $d = 0.995$. Given that allocations under index based designs (and response-adaptive designs more generally) can already be very variable, it does not make sense to choose a discount factor which exacerbates this even further.

A further practical drawback of using a discount factor that is too small is that it will increase the likelihood of the design allocating all patients to only one of the treatments (due to an under exploration). The number of times this occurred out of the 50,000 trial realizations is reported in the "Discarded" column of Table 2. For example, when $d = 0$ and $b = 1$, more than half of the 50,000 trial realizations under $\mathcal{H}_0$ (namely 25,621) resulted in this extreme allocation. Therefore, for the purpose of calculating the test statistic (and hence power) and bias values in these cases, we randomly sampled an observation from the distribution corresponding to the missing arm instead. In contrast, when $d = 0.995$, this problem did not occur in any of the 50,000 trial realizations (and similarly when $d = 0.99$).

Note that all of the aforementioned differences are most pronounced for smaller block sizes (which is when the design is most adaptive) since as the block size grows and the FLGI design becomes more balanced, the respective performance measures eventually converge, irrespective of the value of d.

Overall, provided that d is near to the recommendation suggested by Wang (1991), the performance of the FLGI will be similar — as illustrated by the results for $d = 0.99$ (Table 2) and $d = 0.995$ (Table 3). However, choosing d to be too small in relation to T can alter the behaviour of the design significantly. Moreover, if we were to use this design in a rare disease context, where we envisage it would be best suited, d should be chosen to be large enough so that we account for all of the patient outcomes in the adaptations and hence ensure patient benefit for all.

[Table 2 about here.]

Web Appendix C: Effect of Prior Information on FLGI Performance

In this Web Appendix, we investigate how sensitive the FLGI is to the choice of prior on the location parameter μ_k when the variance is assumed known. Ultimately, the choice of prior on μ_k determines which GI we start the allocation rule with. The minimum amount of information we can assume, a priori, in order to initiate the GI policy is $n_k^0 = 1$ (known variance case) and $n_k^0 = 2$ (unknown variance case) since the GI is undefined for $n_k^0 = 0$. This gives rise to a normal prior with large variance (see Figure 2) which can be used as a so-called 'non-informative' prior (Spiegelhalter et al., 2004, p. 62). All results in the main paper correspond to this 'non-informative' prior so that we can report the effects on patient response and other relevant statistical properties of the FLGI alone, without the influence of additional prior information. However, we now turn our attention to using different priors in conjunction with the FLGI. We use the results for the 'non-informative' prior (in (i) of Table 3) as a reference, and therefore refer to it as a 'reference' prior from hereon.

Taking the two-armed example of Section 3.2 in the main paper (but now assuming known variance), we follow the suggestion provided in Spiegelhalter et al. (2004, Chapter 5) and consider two archetypal priors on μ_1, namely, the *sceptical* and *enthusiastic* prior (with the reference prior on μ_0).

The sceptical prior attempts to formalize the belief that large treatment differences are unlikely. In particular, the sceptical normal prior on μ_1 is centered around the (null hypothesis) value of 0.155 with only a small probability, say 5%, that the true value exceeds the alternative hypothesis value of 0.529, i.e. $\mathcal{P}r(\mu_1 > 0.529) = 0.05$. This corresponds to a prior distribution of $\mu_1 \sim N\left(0.155, \frac{0.64^2}{n_1^0}\right)$, which has the following property

$$-0.64 \times \frac{z_{0.05}}{\sqrt{n_1^0}} = 0.529 - 0.155, \quad (4)$$

where n_1^0 is the implicit (prior) sample size and $z_{0.05} = -1.645$ is the fifth percentile of the standard normal distribution. Rearranging equation (4) gives $n_1^0 \approx 8$. Intuitively, this is

equivalent to having eight patients' worth of information (with null mean) available at the start of the trial, that is, approximately 11% of the trial sample size expressing scepticism and showing no treatment difference. The performance measures of our design when starting with this prior on the experimental arm are shown in (ii) of Table 3 for all block sizes, b.

The enthusiastic prior, on the other hand, is centred on the alternative hypothesis value of 0.529 (with the same variance as the sceptical prior) and specifies that there is little evidence of no treatment effect a priori, i.e. there is a 5% chance of observing a value less than the null mean of 0.155. This corresponds to the following normal prior distribution $\mu_1 \sim N\left(0.529, \frac{0.64^2}{8}\right)$, which is equivalent to having already observed eight 'enthusiastic' responses before the start of the trial. The corresponding results when starting with this prior on the experimental arm are displayed in (iii) of Table 3.

[Table 3 about here.]

Conclusions

The main conclusions to draw from these experiments are that when using the FLGI in the known variance case with a *sceptical* prior on the experimental arm, the power of the design increases whilst the patient benefit measures decrease relative to the corresponding results when starting with the reference prior. This is what we would expect to observe because the sceptical prior implies that there is a 0.95 probability that μ_1 lies below 0.529 (as depicted in Figure 2) which is incorrect under $\mathcal{H}_1$, and as such it provides the FLGI algorithm with a 'false start'. Thus, it takes longer for the design to correctly identify the best arm, resulting in fewer patients allocated to the superior arm but a larger power due to less imbalance.

In contrast, when starting with an *enthusiastic* prior on the experimental arm, the reverse happens (as shown in (iii) of Table 3); the power decreases whilst the patient benefit measures increase (relative to starting with the reference prior). Again, this is not surprising because the enthusiastic prior specifies that the most likely value of μ_1 is 0.529 (as illustrated in Figure 2). Under $\mathcal{H}_1$, this is correct and so it gives the algorithm a 'head start' in the right direction

meaning it identifies the superior arm quicker. Thus, less allocations are made to the control arm resulting in more imbalance and hence reduced power. Under $\mathcal{H}_0$, however, this prior specification on the experimental arm is incorrect and so the FLGI incorrectly allocates fewer patients to the control arm, as observed in (iii) of Table 3 (where the control arm is taken to be the 'superior' arm under $\mathcal{H}_0$). This explains why only $\approx 33\%$ of patients in the trial are allocated to the control arm for all block sizes under $\mathcal{H}_0$. Fewer observations on the control arm leads to an underestimation of $\hat{\mu}_0$ and consequently the treatment effect estimator under $\mathcal{H}_0$ exhibits bias. It is also worth noting that the variability in the allocations decreases when using the enthusiastic prior (relative to the reference prior) since, under $\mathcal{H}_1$, the observed data and prior information match which reduces the uncertainty of the allocations.

Overall, our recommendation is to be very cautious when incorporating prior information into bandit-based designs such as the FLGI because it influences the speed at which the design updates and favors an arm (depending on how informative the prior is). Since these designs are so dynamic anyway, there is not as much to gain from using prior information as there may be with less responsive designs. If the prior specification is correct, then the incoming data will further enhance the effect of the prior and the design will favor the superior arm sooner, whereas if the prior is misspecified, the bandit may spend more time in the exploration phase or degenerate to allocating all patients to one arm. However, it is likely that the incoming data during the trial will eventually dilute the effect of the misspecified prior. How long the design takes to correct for the misspecification depends on the value of n_k^0; the greater its value, the more influence the prior will have. Therefore, if one wishes to use prior information in conjunction with the FLGI, we suggest setting a small value for n_k^0 (as we have in the main paper).

[Figure 2 about here.]

[Table 4 about here.]

References

Edwards, J., Fearnhead, P., and Glazebrook, K. (2017). On the identification and mitigation of weaknesses in the knowledge gradient policy for multi-armed bandits. *Probability in the Engineering and Informational Sciences*, 31(2):239–263.

Gittins, J., Glazebrook, K., and Weber, R. (2011). *Multi-armed Bandit Allocation Indices*. John Wiley & Sons, 2nd edition.

Gittins, J. and Jones, D. (1979). A dynamic allocation index for the discounted multiarmed bandit problem. *Biometrika*, **66**(3):561–565.

Jones, D. (1975). *Search Procedures for Industrial Chemical Research*. PhD thesis, University of Cambridge.

Spiegelhalter, D. J., Abrams, K. R., and Myles, J. P. (2004). *Bayesian Approaches to Clinical Trials and Health-Care Evaluation*. John Wiley & Sons.

Villar, S. S., Bowden, J., and Wason, J. (2015). Response-adaptive randomisation for multi-arm clinical trials using the forward looking Gittins index rule. *Biometrics*, 71(4):969–978.

Wang, Y.-G. (1991). Sequential allocation in clinical trials. *Communications in Statistics - Theory and Methods*, 20(3):791–805.

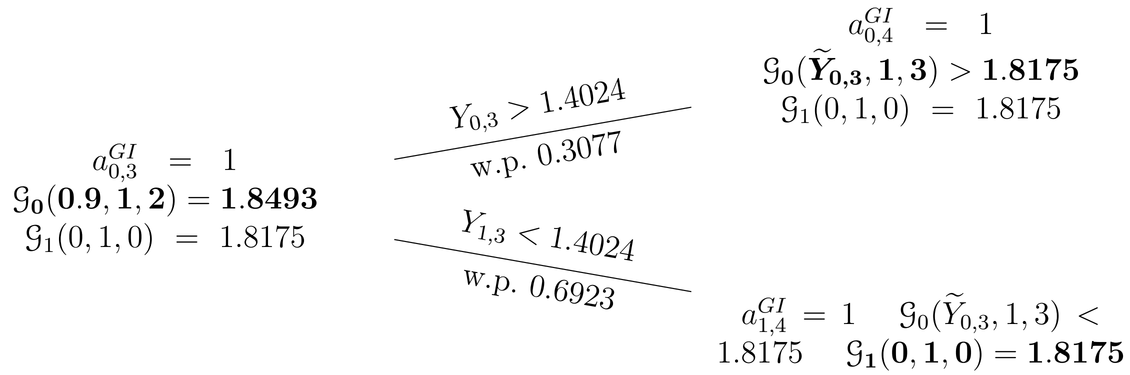

Figure 1. The FLGI rule and a probability tree of all trial histories using the GI rule when $K+1=2$, $b=2$, $d=0.995$ and the state at the start of the second block, $(\tilde{y}_{k,3}, n_k^0 + n_{k,3})$, is $(0.9, 3)$ for arm $k=0$ and $(0,1)$ for arm $k=1$. Bold text indicates the allocated treatment under the GI rule $\{a_{k,t}^{GI}\}$. (Note that for simplicity of the illustration we have omitted the branch corresponding to the case when $Y_{0,3} = 1.4024$ since $\mathcal{P}r(Y_{0,3} = 1.4024) = 0$).

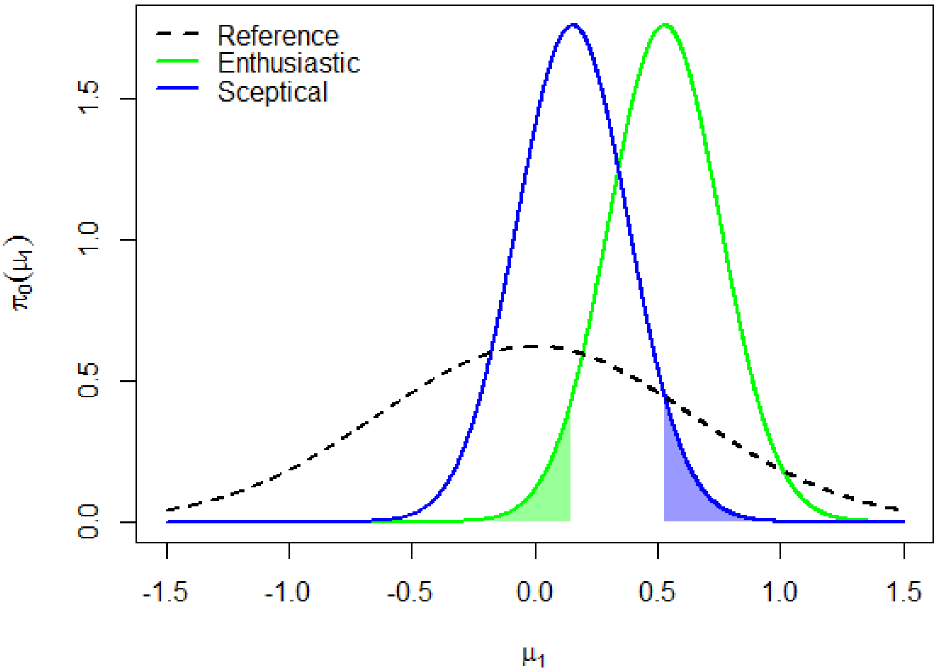

Figure 2. Sceptical and enthusiastic prior densities with the reference prior depicted in black. The sceptics' probability that the true mean response is greater than 0.529 (the alternative value) is 0.05, shown by the purple shaded region. The enthusiasts' probability that the true mean response is less than 0.155 (the null value) is also 0.05, shown by the green shaded region. *NB By 'reference' prior, we refer to the prior containing the least amount of information in order to initiate the Gittins index policy (i.e. a normal prior with large variance).*

$n_k^0 + n_{k,t}$ \ d	0.5	0.6	0.7	0.8	0.9	0.95	0.99	0.995
2	0.23984	1.04741	1.55545	2.81630	5.16921	10.14092	39.33433	65.58475
3	0.15620	0.21476	0.29804	0.43425	0.73571	1.16561	3.10200	4.60490
4	0.09486	0.13001	0.17914	0.25664	0.41606	0.61934	1.34279	1.81263
5	0.07058	0.09673	0.13323	0.19047	0.30608	0.44776	0.90524	1.17299
6	0.05679	0.07791	0.10742	0.15369	0.24666	0.35900	0.70542	0.89632
7	0.04779	0.06564	0.09061	0.12983	0.20866	0.30352	0.59010	0.74336
8	0.04135	0.05685	0.07858	0.11278	0.18165	0.26451	0.51233	0.64259
9	0.03649	0.05021	0.06948	0.09988	0.16128	0.23525	0.45557	0.57012
10	0.03268	0.04500	0.06234	0.08974	0.14527	0.21234	0.41187	0.51498
20	0.01611	0.02228	0.03106	0.04515	0.07444	0.11090	0.22299	0.28120
30	0.01072	0.01485	0.02076	0.03032	0.05049	0.07615	0.15786	0.20137
40	0.00804	0.01115	0.01560	0.02285	0.03829	0.05821	0.12347	0.15903
50	0.00643	0.00892	0.01250	0.01834	0.03086	0.04719	0.10189	0.13229
60	0.00536	0.00744	0.01043	0.01532	0.02586	0.03971	0.08697	0.11368
70	0.00459	0.00638	0.00895	0.01316	0.02225	0.03429	0.07599	0.09991
80	0.00402	0.00558	0.00784	0.01153	0.01953	0.03018	0.06755	0.08927
90	0.00357	0.00496	0.00697	0.01026	0.01741	0.02696	0.06084	0.08077
100	0.00321	0.00447	0.00627	0.00924	0.01570	0.02436	0.05538	0.07381
200	0.00161	0.00224	0.00314	0.00464	0.00793	0.01242	0.02944	0.04024
300	0.00107	0.00149	0.00210	0.00310	0.00531	0.00834	0.02015	0.02790
400	0.00080	0.00112	0.00157	0.00233	0.00399	0.00628	0.01534	0.02142
500	0.00064	0.00090	0.00126	0.00186	0.00319	0.00504	0.01239	0.01740
600	0.00054	0.00075	0.00105	0.00155	0.00266	0.00421	0.01040	0.01466
700	0.00046	0.00064	0.00090	0.00133	0.00228	0.00361	0.00896	0.01268
800	0.00040	0.00056	0.00079	0.00116	0.00200	0.00316	0.00787	0.01117
900	0.00036	0.00050	0.00070	0.00104	0.00178	0.00281	0.00702	0.00999
1000	0.00032	0.00045	0.00063	0.00093	0.00160	0.00253	0.00634	0.00903

Table 1

Gittins indices for a normal reward process with unknown variance where d and $n_k^0 + n_{k,t}$ denote the discount factor and total amount of information, respectively. These values are based on those reported in Table 8.3 of Gittins et al. (2011).

			$\mu_0 = \mu_1 = 0.155$					$\mu_0 = 0.155, \mu_1 = 0.529$			
b	$z_{1-\alpha}$	α	$\mathbb{E}(p^*)$ (s.d.)	RelETO% (s.d.)	Bias (s.d.)	Discarded	$1-\beta$	$\mathbb{E}(p^*)$ (s.d.)	RelETO% (s.d.)	Bias (s.d.)	Discarded
					$d = 0$ (Myopic)						
1	1.827	0.050	0.498 (0.48)	-0.14 (5.45)	0.00 (0.69)	14707	0.213	0.718 (0.43)	23.92 (12.49)	0.08 (0.68)	25621
2	1.829	0.049	0.497 (0.46)	0.02 (5.42)	0.00 (0.60)	7970	0.222	0.761 (0.39)	28.52 (11.53)	0.06 (0.60)	13789
4	1.799	0.050	0.501 (0.43)	0.12 (5.49)	-0.00 (0.50)	2211	0.266	0.810 (0.32)	33.87 (10.13)	0.05 (0.48)	3889
6	1.776	0.050	0.499 (0.41)	-0.24 (5.44)	-0.00 (0.42)	598	0.312	0.832 (0.27)	36.44 (9.03)	0.05 (0.39)	1093
9	1.752	0.050	0.501 (0.38)	0.05 (5.41)	-0.00 (0.33)	94	0.383	0.840 (0.22)	37.12 (8.06)	0.04 (0.31)	174
12	1.720	0.052	0.502 (0.35)	0.46 (5.43)	-0.00 (0.29)	15	0.448	0.837 (0.19)	36.87 (7.44)	0.03 (0.26)	28
18	1.708	0.050	0.499 (0.31)	-0.07 (5.45)	0.00 (0.24)	2	0.533	0.814 (0.15)	34.39 (6.80)	0.02 (0.22)	3
36	1.676	0.050	0.499 (0.21)	0.11 (5.40)	0.00 (0.18)	0	0.694	0.720 (0.10)	24.23 (6.05)	0.00 (0.17)	0
					$d = 0.5$						
1	1.819	0.050	0.497 (0.46)	0.02 (5.42)	0.00 (0.66)	4894	0.228	0.772 (0.39)	29.64 (11.38)	0.07 (0.66)	18359
2	1.818	0.049	0.501 (0.44)	0.03 (5.45)	-0.00 (0.58)	2637	0.243	0.804 (0.35)	33.38 (10.54)	0.07 (0.58)	9769
4	1.770	0.054	0.497 (0.42)	-0.15 (5.47)	0.00 (0.49)	669	0.287	0.832 (0.29)	36.20 (9.45)	0.07 (0.46)	2696
6	1.765	0.052	0.499 (0.40)	-0.25 (5.45)	-0.00 (0.41)	192	0.329	0.844 (0.25)	37.72 (8.60)	0.06 (0.38)	763
9	1.757	0.049	0.501 (0.37)	0.14 (5.45)	-0.00 (0.33)	24	0.391	0.844 (0.21)	37.41 (7.80)	0.05 (0.30)	101
12	1.749	0.048	0.500 (0.34)	-0.05 (5.39)	-0.00 (0.29)	3	0.441	0.838 (0.18)	37.13 (7.26)	0.04 (0.26)	25
18	1.707	0.050	0.503 (0.30)	-0.17 (5.43)	-0.00 (0.24)	0	0.543	0.816 (0.15)	34.62 (6.70)	0.03 (0.22)	0
36	1.677	0.049	0.501 (0.21)	-0.16 (5.43)	-0.00 (0.18)	0	0.693	0.720 (0.10)	24.04 (6.09)	0.01 (0.17)	0
					$d = 0.99$						
1	1.981	0.050	0.498 (0.35)	0.08 (5.43)	0.00 (0.53)	0	0.209	0.882 (0.19)	41.94 (7.38)	0.22 (0.47)	0
2	1.944	0.051	0.502 (0.34)	0.08 (5.44)	-0.00 (0.47)	0	0.255	0.879 (0.17)	41.44 (7.20)	0.19 (0.42)	0
4	1.907	0.051	0.501 (0.32)	-0.31 (5.45)	-0.00 (0.41)	0	0.313	0.871 (0.16)	40.62 (6.91)	0.14 (0.36)	0
6	1.885	0.049	0.500 (0.31)	-0.20 (5.41)	-0.00 (0.36)	0	0.349	0.865 (0.15)	39.95 (6.73)	0.11 (0.31)	0
9	1.850	0.049	0.501 (0.29)	0.36 (5.44)	-0.00 (0.31)	0	0.409	0.851 (0.14)	38.38 (6.64)	0.08 (0.26)	0
12	1.816	0.051	0.499 (0.28)	0.16 (5.42)	0.00 (0.27)	0	0.467	0.839 (0.13)	37.11 (6.44)	0.06 (0.23)	0
18	1.758	0.050	0.499 (0.25)	-0.10 (5.44)	0.00 (0.23)	0	0.556	0.811 (0.12)	33.89 (6.31)	0.04 (0.20)	0
36	1.684	0.051	0.499 (0.19)	-0.03 (5.44)	0.00 (0.18)	0	0.710	0.716 (0.10)	23.45 (6.02)	0.01 (0.17)	0

Table 2

The effect of altering the discount factor, d, on the performance of the FLGI for a two-armed trial when $\sigma_k^2 = 0.64^2$ is assumed known and $T = 72$, averaged over 50,000 trial replications. NB The "Discarded" column reports the number of trials that resulted in an extreme allocation with all patients being allocated to only one arm.

			$\mu_0 = \mu_1 = 0.155$					$\mu_0 = 0.155, \mu_1 = 0.529$	
b	$z_{1-\alpha}$	α	$\mathbb{E}(p^*)$ (s.d.)	Rel$ETO\%$ (s.d.)	Bias (s.d.)	$1-\beta$	$\mathbb{E}(p^*)$ (s.d.)	Rel$ETO\%$ (s.d.)	Bias (s.d.)
			(i) Reference ($n_0^0 = 1$) vs. Reference ($n_1^0 = 1$)						
1	1.991	0.053	0.500 (0.33)	-0.06 (5.42)	-0.00 (0.49)	0.229	0.882 (0.16)	41.69 (7.01)	0.22 (0.45)
2	1.969	0.051	0.500 (0.32)	0.34 (5.42)	-0.00 (0.44)	0.270	0.878 (0.16)	41.27 (6.85)	0.19 (0.41)
4	1.949	0.046	0.502 (0.30)	0.32 (5.43)	-0.00 (0.38)	0.313	0.870 (0.14)	40.30 (6.65)	0.15 (0.35)
6	1.911	0.048	0.499 (0.29)	-0.03 (5.45)	0.00 (0.34)	0.360	0.861 (0.14)	39.38 (6.59)	0.11 (0.30)
9	1.864	0.049	0.498 (0.28)	-0.15 (5.43)	0.00 (0.30)	0.423	0.848 (0.13)	38.14 (6.53)	0.08 (0.26)
12	1.825	0.050	0.502 (0.26)	0.11 (5.42)	-0.00 (0.27)	0.478	0.834 (0.13)	36.68 (6.41)	0.06 (0.23)
18	1.766	0.051	0.502 (0.24)	0.14 (5.44)	-0.00 (0.23)	0.565	0.807 (0.12)	33.72 (6.30)	0.04 (0.20)
36	1.682	0.050	0.500 (0.19)	0.26 (5.45)	0.00 (0.17)	0.712	0.714 (0.09)	23.25 (5.98)	0.01 (0.17)
			(ii) Reference ($n_0^0 = 1$) vs. Sceptical ($n_1^0 = 8$)						
1	2.004	0.051	0.470 (0.32)	0.25 (5.44)	0.07 (0.43)	0.427	0.844 (0.18)	37.72 (6.51)	0.22 (0.41)
2	1.953	0.050	0.479 (0.31)	-0.37 (5.43)	0.05 (0.38)	0.491	0.833 (0.17)	36.22 (6.46)	0.17 (0.34)
4	1.913	0.050	0.484 (0.29)	-0.21 (5.43)	0.04 (0.33)	0.538	0.820 (0.17)	34.96 (6.38)	0.13 (0.29)
6	1.886	0.049	0.491 (0.28)	-0.49 (5.43)	0.03 (0.31)	0.565	0.811 (0.16)	33.92 (6.44)	0.10 (0.26)
9	1.856	0.051	0.494 (0.27)	-0.26 (5.45)	0.02 (0.28)	0.590	0.800 (0.16)	32.92 (6.40)	0.08 (0.24)
12	1.844	0.049	0.497 (0.26)	0.27 (5.43)	0.02 (0.25)	0.597	0.788 (0.15)	31.44 (6.41)	0.07 (0.22)
18	1.792	0.051	0.499 (0.24)	-0.20 (5.42)	0.02 (0.22)	0.650	0.771 (0.14)	29.61 (6.32)	0.05 (0.20)
36	1.715	0.052	0.483 (0.18)	0.24 (5.43)	0.01 (0.18)	0.710	0.717 (0.12)	23.69 (6.09)	0.02 (0.17)
			(iii) Reference ($n_0^0 = 1$) vs. Enthusiastic ($n_1^0 = 8$)						
1	1.964	0.047	0.315 (0.24)	-0.00 (5.41)	0.14 (0.37)	0.176	0.920 (0.08)	45.86 (5.79)	0.24 (0.41)
2	1.908	0.052	0.323 (0.24)	-0.44 (5.45)	0.13 (0.34)	0.234	0.911 (0.08)	44.80 (5.83)	0.20 (0.36)
4	1.892	0.049	0.329 (0.23)	-0.02 (5.42)	0.11 (0.31)	0.275	0.902 (0.09)	43.88 (5.82)	0.17 (0.33)
6	1.872	0.050	0.332 (0.22)	0.24 (5.41)	0.10 (0.30)	0.313	0.896 (0.09)	43.38 (5.86)	0.15 (0.31)
9	1.864	0.048	0.337 (0.22)	0.15 (5.39)	0.09 (0.28)	0.348	0.887 (0.09)	42.32 (5.86)	0.13 (0.29)
12	1.834	0.052	0.338 (0.21)	-0.10 (5.39)	0.08 (0.27)	0.388	0.878 (0.09)	41.37 (5.87)	0.12 (0.28)
18	1.804	0.054	0.338 (0.20)	-0.19 (5.45)	0.07 (0.25)	0.444	0.865 (0.10)	39.83 (5.95)	0.10 (0.25)
36	1.761	0.050	0.324 (0.17)	0.08 (5.41)	0.05 (0.21)	0.515	0.836 (0.11)	36.62 (6.07)	0.05 (0.21)

Table 3

The effect of using archetypal priors on the performance of the FLGI for a two-armed trial when $\sigma_k^2 = 0.64^2$ is assumed known, $T = 72$ and $d = 0.995$, averaged over 50,000 trial replications.

	$\mu_0 = \mu_1 = \mu_2 = -0.05$					$\mu_0 = -0.05, \mu_1 = 0.07, \mu_2 = 0.13$			
Design	$t_{1-\alpha}$	α	$\mathbb{E}(p^*)$ (s.d.)	RelETO% (s.d.)	Bias (s.d.)	$1-\beta$	$\mathbb{E}(p^*)$ (s.d.)	RelETO% (s.d.)	Bias (s.d.)
**ER** ($b = 1$)	1.595	0.0997	0.3332 (0.04)	-0.28 (3.79)	-0.0007 (0.08)	0.7608	0.3333 (0.04)	-0.07 (3.87)	-0.0002 (0.08)
**FLGI**									
$b = 1$	1.731	0.0992	0.3331 (0.20)	0.03 (3.77)	0.0006 (0.12)	0.4936	0.6122 (0.23)	88.13 (4.34)	0.0212 (0.13)
$b = 2$	1.718	0.0994	0.3336 (0.20)	-0.09 (3.79)	0.0002 (0.12)	0.5121	0.6087 (0.22)	87.48 (4.35)	0.0209 (0.13)
$b = 4$	1.737	0.0979	0.3343 (0.19)	0.10 (3.79)	0.0001 (0.12)	0.5100	0.6024 (0.22)	86.04 (4.35)	0.0203 (0.13)
$b = 8$	1.741	0.0954	0.3323 (0.19)	-0.47 (3.79)	-0.0003 (0.11)	0.5235	0.5938 (0.21)	83.52 (4.35)	0.0198 (0.12)
$b = 15$	1.709	0.1018	0.3337 (0.18)	-0.03 (3.79)	0.0001 (0.11)	0.5582	0.5824 (0.21)	80.94 (4.34)	0.0191 (0.12)
$b = 20$	1.725	0.1001	0.3342 (0.18)	0.00 (3.78)	0.0004 (0.11)	0.5608	0.5716 (0.20)	77.82 (4.32)	0.0166 (0.11)
$b = 40$	1.662	0.1009	0.3342 (0.17)	-0.35 (3.80)	-0.0004 (0.10)	0.6100	0.5296 (0.18)	66.48 (4.26)	0.0047 (0.10)
$b = 60$	1.591	0.1009	0.3337 (0.15)	-0.05 (3.80)	0.0004 (0.09)	0.6697	0.4835 (0.16)	51.91 (4.16)	-0.0013 (0.09)
**FLGI-HZ** ($\gamma = 2$)									
$b = 1$	1.603	0.0972	0.3333 (0.04)	0.37 (3.79)	-0.0001 (0.08)	0.6157	0.5137 (0.05)	67.11 (3.84)	-0.0001 (0.10)
$b = 2$	1.592	0.0996	0.3334 (0.04)	-0.41 (3.80)	-0.0002 (0.08)	0.6161	0.5137 (0.05)	67.19 (3.85)	-0.0005 (0.10)
$b = 4$	1.587	0.1009	0.3332 (0.04)	0.08 (3.79)	0.0000 (0.08)	0.6209	0.5134 (0.05)	66.96 (3.84)	0.0002 (0.10)
$b = 8$	1.600	0.0979	0.3331 (0.04)	0.79 (3.79)	-0.0002 (0.08)	0.6162	0.5133 (0.05)	67.03 (3.84)	-0.0004 (0.10)
$b = 15$	1.585	0.0994	0.3332 (0.04)	-0.18 (3.80)	-0.0006 (0.08)	0.6207	0.5131 (0.05)	66.86 (3.85)	-0.0002 (0.10)
$b = 20$	1.605	0.0991	0.3334 (0.04)	-0.32 (3.80)	-0.0001 (0.08)	0.6187	0.5115 (0.05)	66.74 (3.87)	-0.0001 (0.10)
$b = 40$	1.587	0.1014	0.3334 (0.04)	0.29 (3.80)	-0.0002 (0.08)	0.6839	0.4827 (0.06)	53.2 (3.93)	0.0003 (0.09)
$b = 60$	1.598	0.1000	0.3335 (0.05)	0.25 (3.79)	0.0004 (0.08)	0.7665	0.4240 (0.05)	19.34 (4.08)	0.0000 (0.08)
**CFLGI**									
$b = 1$	1.530	0.1012	0.2894 (0.16)	-0.01 (3.80)	-0.0236 (0.10)	0.7254	0.4780 (0.18)	30.41 (4.12)	-0.0171 (0.09)
$b = 2$	1.533	0.1009	0.2922 (0.16)	0.11 (3.78)	-0.0223 (0.10)	0.7272	0.4756 (0.17)	30.28 (4.12)	-0.0164 (0.09)
$b = 4$	1.520	0.1017	0.2949 (0.16)	-0.06 (3.79)	-0.0209 (0.10)	0.7324	0.4734 (0.17)	30.20 (4.09)	-0.0157 (0.09)
$b = 8$	1.522	0.1027	0.2960 (0.15)	-0.55 (3.80)	-0.0203 (0.10)	0.7366	0.4685 (0.17)	29.11 (4.07)	-0.0144 (0.09)
$b = 15$	1.530	0.0988	0.2976 (0.15)	0.04 (3.80)	-0.0190 (0.10)	0.7361	0.4615 (0.16)	27.68 (4.09)	-0.0133 (0.09)
$b = 20$	1.534	0.1020	0.2981 (0.15)	-0.53 (3.80)	-0.0180 (0.09)	0.7328	0.4554 (0.16)	26.79 (4.07)	-0.0130 (0.09)
$b = 40$	1.538	0.1006	0.3029 (0.14)	0.20 (3.78)	-0.0134 (0.09)	0.7425	0.4331 (0.14)	20.98 (4.03)	-0.0105 (0.08)
$b = 60$	1.540	0.1005	0.3077 (0.13)	-0.26 (3.79)	-0.0082 (0.08)	0.7552	0.4094 (0.12)	15.80 (4.02)	-0.0070 (0.08)
**TP**									
$b = 1$	1.582	0.1009	0.3116 (0.09)	0.11 (3.78)	-0.0127 (0.09)	0.7822	0.3403 (0.06)	-5.75 (3.82)	-0.0019 (0.08)
$b = 2$	1.580	0.0991	0.3119 (0.09)	0.08 (3.80)	-0.0128 (0.09)	0.7791	0.3402 (0.05)	-6.68 (3.82)	-0.0028 (0.08)
$b = 4$	1.571	0.0997	0.3121 (0.09)	0.05 (3.79)	-0.0128 (0.09)	0.7824	0.3400 (0.05)	-5.57 (3.81)	-0.0019 (0.08)
$b = 8$	1.579	0.0991	0.3130 (0.09)	-0.33 (3.78)	-0.0121 (0.09)	0.7820	0.3393 (0.05)	-6.07 (3.83)	-0.0016 (0.08)
$b = 15$	1.570	0.1009	0.3148 (0.08)	0.45 (3.78)	-0.0107 (0.09)	0.7793	0.3381 (0.05)	-6.31 (3.84)	-0.0023 (0.08)
$b = 20$	1.576	0.0982	0.3146 (0.08)	-0.34 (3.79)	-0.0098 (0.08)	0.7781	0.3370 (0.05)	-6.02 (3.82)	-0.0020 (0.08)
$b = 40$	1.567	0.1042	0.3174 (0.08)	-0.11 (3.80)	-0.0069 (0.08)	0.7792	0.3331 (0.05)	-6.68 (3.91)	-0.0013 (0.08)
$b = 60$	1.574	0.1021	0.3131 (0.07)	-0.35 (3.78)	-0.0051 (0.08)	0.7738	0.3214 (0.06)	-11.58 (3.95)	-0.0011 (0.08)
**TS**									
$b = 1$	1.629	0.1024	0.3331 (0.09)	0.09 (3.78)	0.0002 (0.09)	0.7313	0.4589 (0.10)	47.23 (4.08)	0.0141 (0.10)
$b = 2$	1.651	0.0985	0.3340 (0.09)	-0.10 (3.78)	0.0009 (0.09)	0.7223	0.4574 (0.10)	46.67 (4.07)	0.0132 (0.10)
$b = 4$	1.641	0.0986	0.3337 (0.09)	0.18 (3.80)	0.0006 (0.09)	0.7267	0.4557 (0.10)	46.10 (4.08)	0.0138 (0.10)
$b = 8$	1.620	0.1028	0.3336 (0.08)	0.10 (3.79)	0.0000 (0.09)	0.7366	0.4513 (0.10)	44.76 (4.07)	0.0124 (0.09)
$b = 15$	1.626	0.1012	0.3327 (0.08)	0.38 (3.78)	-0.0005 (0.09)	0.7349	0.4443 (0.09)	43.38 (4.07)	0.0121 (0.09)
$b = 20$	1.632	0.1006	0.3330 (0.08)	0.01 (3.78)	-0.0001 (0.09)	0.7344	0.4390 (0.09)	41.59 (4.06)	0.0110 (0.09)
$b = 40$	1.635	0.0980	0.3329 (0.07)	0.00 (3.79)	-0.0003 (0.08)	0.7410	0.4191 (0.08)	34.82 (4.05)	0.0093 (0.09)
$b = 60$	1.609	0.1016	0.3328 (0.07)	0.03 (3.78)	-0.0006 (0.08)	0.7478	0.3979 (0.08)	27.29 (4.02)	0.0055 (0.09)
**Gwise**									
$n^{\mathrm{ER}} = 2$	1.778	0.0998	0.3343 (0.08)	0.50 (3.82)	0.0003 (0.09)	0.7101	0.3336 (0.08)	0.12 (4.12)	0.0002 (0.09)
$n^{\mathrm{ER}} = 4$	1.620	0.1011	0.3334 (0.05)	-0.32 (3.78)	0.0001 (0.08)	0.7599	0.3328 (0.05)	-0.13 (3.89)	-0.0006 (0.08)
$n^{\mathrm{ER}} = 8$	1.618	0.1010	0.3332 (0.04)	-0.09 (3.78)	-0.0001 (0.08)	0.7627	0.3332 (0.04)	-0.01 (3.9)	-0.0003 (0.08)

Table 4

Comparison of performance measures for a three-armed trial using different designs when the variance is assumed unknown and $T = 120$, averaged over 50,000 trial replications. Note that the true variance of the response is $\sigma_k^2 = 0.346^2$ for $k \in \{0, 1, 2\}$.
