# Supplementary material for: A response‐adaptive randomization procedure for multi‐armed clinical trials with normally distributed outcomes
Source: Biometrics. 2019 Sep 19;76(1):197–209. doi: 10.1111/biom.13119 (PMC7078926; doi:10.1111/biom.13119)
Supplement: Supplementary file 3 — Supplementary Information [file BIOM-76-197-s003.pdf]

Supporting Information

Web Appendices may be found in the Supporting Information available with this article on the Wiley Online Library. R code to implement the FLGI method can also be found here.
